# Inflammatory Manifestations, Therapeutic Interventions, and Cancer Risk in Psoriasis: Current Epidemiological and Mechanistic Evidence

**DOI:** 10.3390/ijms27156780

**Published:** 2026-07-29

**Authors:** Aikaterini Lymperi, Evgenia Lamprianidou, Theodora Adamantidi, Maria Chatzikamari, Nikolaos Loizidis, Vassiliki Dania, Alexandros Tsoupras

**Affiliations:** 1 Hephaestus Laboratory, School of Chemistry, Faculty of Sciences, Democritus University of Thrace, Kavala University Campus, 65404 Kavala, Greece; ailyber@chem.duth.gr (A.L.); evfabri@chem.duth.gr (E.L.); theodoradamantidi@gmail.com (T.A.); 2 Hematology Department, General Hospital of Kavala, St Sillas, 65500 Kavala, Greece; xatzifil@gmail.com; 3 Rheumatology Department, General Hospital of Kavala, St Sillas, 65500 Kavala, Greece; nikloizidis@gmail.com; 4 First Department of Orthopedic Surgery, School of Medicine, National and Kapodistrian University of Athens, ATTIKON University General Hospital, 12462 Athens, Greece; viky_dania@yahoo.gr

**Keywords:** psoriasis, cancer risk, autoimmune diseases, psoriatic treatment, biological therapies, molecular link, inflammation, cardiovascular disease

## Abstract

Psoriasis is a chronic, autoimmune skin disease driven by persistent inflammation and immune dysfunction that severely impairs quality of life and is associated with serious comorbidities, including psoriatic arthritis, cardiovascular diseases (CVDs), and depression. Emerging epidemiological evidence suggests an association between psoriasis and an increased risk of certain malignancies, such as breast cancer (BC) and non-Hodgkin’s lymphoma (NHL), although the strength and consistency of these associations vary across study designs, and the underlying thrombo-inflammatory mechanisms remain incompletely understood. Several therapeutic approaches, including topical therapies, conventional systemic drugs (e.g., methotrexate and cyclosporine), and biological agents have been investigated for their potential associations with malignancy risk. However, the available evidence is heterogeneous and influenced by disease severity, treatment duration, cumulative exposure, and patient-related confounding factors. While some epidemiological studies have reported associations between conventional therapies and selected skin or hematological malignancies, combined or sequential treatment regimens further complicate the interpretation of treatment-related cancer risk. Similarly, Janus kinase (JAK) inhibitors have been associated with higher reported rates of lymphoma and non-melanoma skin cancer than tumor necrosis factor α inhibitors (TNFi-α) in some observational studies. Recent studies also highlight the clinical utility of inflammatory markers, specifically the neutrophil-to-lymphocyte ratio (NLR), platelet-to-lymphocyte (PLR) ratio, and systemic immune-inflammation index (SII), for monitoring systemic inflammation and treatment response, while certain therapies may additionally influence CVD risk. Despite these advances, substantial heterogeneity across observational studies, meta-analyses, and Mendelian randomization analyses preclude definitive conclusions regarding causality. Overall, this review synthesizes the current epidemiological and mechanistic evidence linking psoriasis, chronic inflammation, therapeutic interventions, cancer risk, and cardiovascular comorbidities, while highlighting the need for large prospective studies and standardized analytical approaches.

## 1. Introduction

Autoimmune diseases (AIDs) comprise a heterogeneous group of chronic disorders characterized by a breakdown of immune tolerance and leading to immune-mediated tissue damage [1], evidenced by an excessive host immune response from impaired immune regulation [2]. More than 80 different AIDs have been described [3], collectively affecting multiple organs and organ-specific conditions and approximately 3–10% of the global population, especially in Western societies [2,4]. Common examples include psoriasis (prevalence: 2–3%), rheumatoid arthritis (RA, prevalence: 0.5–1%) and ankylosing spondylitis (AS, prevalence: 0.3%) [2,4]. Epidemiological data also suggest that women exhibit heightened predispositions to AIDs. This is largely attributed to endocrine fluctuations across their lifespan (e.g., puberty, pregnancy, and menopause).These hormonal shifts modulate immune regulation and may promote pro-inflammatory responses or autoreactivity and thereby contribute to AIDs pathogenesis [2,4].

Immune dysfunction plays a critical role not only in the development of AIDs but also in oncogenesis. Accumulating evidence indicates an association between AIDs and malignancies, as well as various comorbidities that substantially impair patient quality of life [1,5]. Supporting this association, a comprehensive review documented positive associations between 23 distinct AIDs and carcinogenesis, although some studies have yielded mixed results [4]. Psoriasis is one of the most prevalent immune-mediated inflammatory diseases and is increasingly recognized as a systemic disorder related to multiple comorbidities like cardiovascular diseases (CVDs) and malignancy [1,5].

Psoriasis is a chronic, systemic inflammatory disease primarily affecting the skin [6]. Although psoriasis may occur anywhere on the body, it primarily affects the scalp, elbows, knees and joints [7]. Clinically, psoriatic patients present erythematous plaques covered by silvery-white scales resulting from the hyperproliferation and impaired differentiation of keratinocytes, accompanied by induced irritation, pruritus, and pain [7,8,9]. This process results in fissuring, skin peeling—particularly pronounced on the scalp—inflammation, and physical pain [7]. The etiology of psoriasis involves genetic and environmental susceptibility, though its exact cause has not been fully elucidated [8]. Psoriasis affects individuals globally, irrespective of geographical distribution, age, or biological sex, but it is most commonly observed between the ages of 15 and 35 years and 50 and 65 years [6,8].

The pathogenesis of psoriasis is driven by an overactivation of the adaptive and innate immune systems, leading immune cells to attack healthy cutaneous tissues [8]. This autoreactive immune profile supports the classification of psoriasis as an immune-mediated inflammatory disease with autoimmune characteristics [8]. Mechanistically, the life cycle of keratinocytes is markedly accelerated, reducing from a physiological 28 days to 3–6 days, which precipitates the rapid accumulation of cornified cells on the epidermal surface [8]. Disease onset or exacerbation can be triggered by acute physiological stress or preceding microbial infections (e.g., *Streptococcus* species), and clinical symptoms can be further aggravated by lifestyle and environmental factors such as cold, dry climates, smoking, obesity, or a susceptible genetic background [8,10].

Current understanding of psoriasis pathogenesis centers on the IL-23/Th17/IL-17 immune axis. Following environmental or genetic triggers, plasmacytoid dendritic cells (DCs) initiate innate immune activation through the production of type I interferons (IFNs), promoting the activation of myeloid DCs. These cells subsequently release IL-23 and TNF-α, driving the differentiation and expansion of Th17 lymphocytes. Th17-derived cytokines, particularly IL-17A, IL-17F, and IL-22, stimulate keratinocyte hyperproliferation, impair epidermal differentiation, and amplify the production of additional inflammatory mediators, thereby establishing a self-perpetuating inflammatory loop. Persistent activation of this cytokine network contributes not only to chronic cutaneous inflammation but also to the low-grade systemic inflammatory state that underlies many psoriasis-associated CVDs and oncological comorbidities [11,12].

While a definitive cure for psoriasis remains elusive, a broad spectrum of therapeutic interventions is available to manage symptoms and achieve clinical remission, with treatment selection dictated by disease severity [6]. Therapeutic interventions range from topical therapies, like corticosteroid creams or vitamin D_3_ analogues for mild cases, to systemic therapies such as conventional immunomodulators (e.g., methotrexate and cyclosporine), systemic corticosteroids, or targeted biological agents for moderate-to-severe conditions [6]. Psoriasis is strongly associated with the development of multiple systemic comorbidities, including psoriatic arthritis, CVDs, diabetes, obesity, and clinical depression, which collectively exert a severe psychological and physical burden on the patient [6,7,11]. These comorbidities are thought to arise from persistent low-grade systemic inflammation, sustained activation of the IL-23/Th17/IL-17 axis, endothelial dysfunction, and thrombo-inflammatory signaling that extend beyond the skin and contribute to systemic disease [11,12].

**Figure 1:**
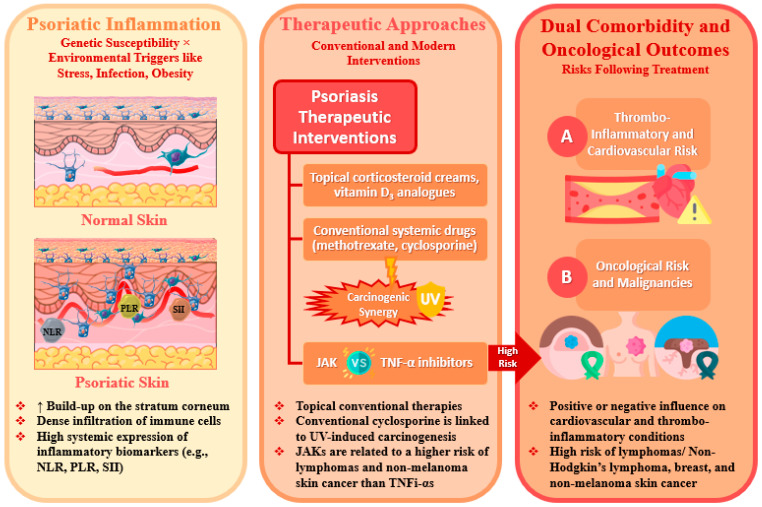
The immuno-inflammatory crossroads of psoriasis, therapeutics, and comorbid risks. The schematic illustrates three integrated axes: (**Left**) the transition from normal to psoriatic skin driven by gene–environment interactions, characterized by stratum corneum thickening, dense immune cell infiltration, and elevated systemic inflammatory biomarkers; (**Center**) conventional and modern therapeutic interventions, highlighting carcinogenic synergies (e.g., cyclosporine and UV exposure) and relative risk differences between drug classes (e.g., JAKis vs. TNF-is); and (**Right**) the resulting clinical outcomes including (**A**) thrombo-inflammatory and cardiovascular risks and (**B**) elevated oncological risks. (Parts of this figure were obtained from the free online databases https://smart.servier.com/ and https://www.flaticon.com/).

Within this review, the pathophysiological link between psoriasis-induced inflammation and various metabolic conditions and CVDs will be examined. Furthermore, the inflammatory bridge connecting psoriasis and its current therapeutic strategies to different malignancies will be reviewed at the molecular, cellular, and epidemiological level. Figure 1 highlights the main key points of this review.

## 2. Literature Research Strategy

This narrative review was informed by a structured literature search conducted exclusively using the Scopus database to identify recent evidence regarding the relationship between psoriasis, inflammation, therapeutic approaches, and cancer risk. The search primarily focused on original research articles published between 2019 and 2024, written in English, and available as open-access publications. Open-access articles were prioritized to ensure full-text availability for comprehensive evaluation and to facilitate reproducibility. The authors acknowledge that this approach may have excluded relevant subscription-based publications and represents a limitation of the review.

Titles and abstracts were initially screened for relevance, followed by full-text assessment of potentially eligible studies. Studies were selected based on their relevance to the review objectives, methodological quality, and availability of sufficient epidemiological or mechanistic data regarding psoriasis, inflammation, cancer risk, CVDs, or therapeutic interventions. Because of the considerable heterogeneity in study design, populations, and reported outcomes, a formal meta-analysis was not performed and the evidence was synthesized narratively.

Searches were performed using the Boolean OR/AND operators and multiple combinations of keywords related to AIDs, psoriasis, cancer, malignancy, neoplasms, oncogenesis, inflammation, thrombo-inflammatory mechanisms, cardiovascular disease, therapeutic interventions, and associated risk factors. Representative search terms comprised combinations of “autoimmune disease”, “psoriasis”, “cancer”, “malignancy”, “neoplasm”, “cancer risk”, “association”, “thrombosis”, “platelets”, “platelet-activating factor”, “lifestyle”, and “inflammation”. Additional indexed keywords, such as “article”, “autoimmune disease”, “human”, “adult”, and “cancer risk” were incorporated when appropriate to improve the specificity of the retrieved literature.

The literature search was conducted in two sequential phases. The first phase focused on the epidemiological relationship between psoriasis and malignancy. Following duplicate article removal and relevance assessment, 33 original articles were retained and categorized into two broad thematic categories: (i) studies evaluating the baseline predisposition to cancer in patients with psoriasis and (ii) studies investigating the association between oncogenesis and the administration of systemic psoriatic treatments. These studies were subsequently organized according to malignancy type and therapeutic class to facilitate a structured presentation of available evidence.

The second phase focused on the mechanistic links between psoriasis-induced inflammation and oncogenesis. Additional searches were performed using combinations of terms related to thrombosis, thromboembolisms, platelet biology, platelet-activating factor, inflammation, lifestyle interventions, and CVDs. Following duplicate article removal and relevance assessment, 50 original articles were selected. These publications were grouped into thematic categories addressing: (i) inflammatory mechanisms underlying psoriasis pathogenesis, (ii) modulation of inflammatory pathways by anti-psoriatic treatments, (iii) interactions between chronic inflammation and CVDs, (iv) lifestyle or natural therapeutic approaches that may influence systemic inflammation, and (v) thrombo-inflammatory mechanisms including thrombosis and thromboembolism.

To provide a comprehensive overview of the topic, additional supporting studies addressing CVDs and selected preclinical in vivo investigations employing psoriasis-like murine models were incorporated where they provided important mechanistic insights relevant to the objectives of this review.

**Figure 2:**
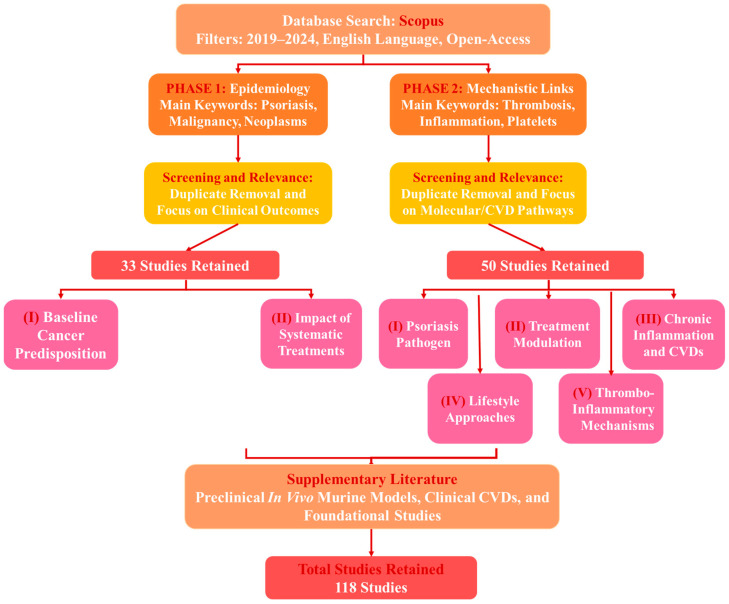
Conceptual flowchart of the literature search and thematic study selection process. This narrative framework, outlines the parallel, phase-driven retrieval strategy used to isolate epidemiological associations (Phase 1, n = 33) and mechanistic pathways (Phase 2, n = 50) from the Scopus database, supplemented by targeted preclinical and cardiovascular comorbidity references.

Given the broad scope and objectives of this narrative review, a structured literature search was employed to identify and synthesize the most relevant evidence. Consequently, formal systematic review procedures, including PRISMA-guided study selection and risk-of-bias assessment, were not applied. A flowchart summarizing the literature search and study selection process is presented in Figure 2.

## 3. Inflammation Associated with the Autoimmune Manifestations of Psoriasis

Psoriasis, as a systemic chronic inflammatory disease, is closely associated with the continuous activation of the immune and hemostatic systems, contributing to an increased risk of thrombo-inflammatory complications [13]. Cutaneous and systemic inflammation in psoriasis is characterized by the upregulated expression of pro-inflammatory cytokines, such as tumor necrosis factor α (TNF-α), interleukin-1β (IL-1β), and interferon-γ (IFN-γ), which directly contribute to endothelial dysfunction [13]. Specifically, TNF-α and IL-1β induce the overexpression of tissue factor (TF) in endothelial cells at both the mRNA and protein levels, promoting platelet activation and disrupting intravascular hemostasis [14]. This cascade significantly escalates the risk of atherothrombosis [14].

TF serves as the primary cellular receptor and cofactor for coagulation factor VII/VIIa (FVII/FVIIa). The resultant TF-FVIIa complex acts as the principal initiator of the extrinsic coagulation pathway, driving the pathological progression of arterial and venous thrombosis [15]. In parallel, systemic inflammation in psoriasis enhances thrombin production and endothelial TF expression, establishing a self-perpetuating positive feedback loop between inflammation and thrombosis [13]. While TNF-α and IL-1β exacerbate this prothrombotic endothelial phenotype, IFN-γ despite upregulating other synergistic cytokines, does not appear to directly stimulate TF production or acute thrombus formation [14]. Furthermore, psoriasis is associated with elevated metabolic homocysteine and reduced vitamin B_12_ levels, which can compound endothelial injury via oxidative stress, further accelerating thrombogenesis [16,17]. The clinical importance of these mechanisms is underscored by a documented increase in the incidence of thromboembolic events among psoriatic patients, further reinforcing the critical need to understand the inflammatory–thrombotic axis and its interactions [13,14,16].

Although the role of cytokine-driven endothelial activation and TF expression is well supported by experimental and translational studies, direct evidence linking these mechanisms to thromboembolic events in psoriasis is derived mainly from observational human studies. Consequently, while the inflammatory thrombotic axis is considered a biologically plausible contributor to CVDs, the contribution of each individual cytokine pathway to long-term clinical outcomes remains incompletely defined [16,17].

Dendritic cells (DCs), functioning as professional antigen-presenting cells, are activated by pathogen- or damage-related molecular motifs (PAMPs/DAMPs) through pattern recognition receptors (PRRs). Upon activation, they undergo phenotypic maturation and migrate to regional lymph nodes to prime naïve T cells [18]. The systemic trafficking of DCs depends on the upregulation of specific chemokine receptors (CCRs), principally CCR7, which coordinates their migration and downstream interaction with T lymphocytes [18]. Additionally, chemokines such as CCL20, secreted by activated keratinocytes within cutaneous lesions, actively recruit CCR6+ monocytes and promote the accumulation of inflammatory DCs in the skin, where they aggravate local inflammation [18,19].

These inflammatory DCs release key interleukins, including IL-12 and IL-23, which drive the differentiation and clonal expansion of helper T17 (Th17) cells. The subsequent secretion of effector cytokines, such as IL-17 and IL-22, impairs physiological keratinocyte function and precipitates abnormal epidermal thickening [18]. The direct involvement of DCs in modulating inducible skin-associated lymphoid tissue (iSALT) architectures within psoriatic plaques reinforces their status as pivotal regulators in propagating and maintaining chronic tissue inflammation [18]. The complexity of pathophysiological pathways, characterized by tight feedback loops between immune cell subsets, cytokines and chemokines, positions DCs as promising therapeutic targets for next-generation immunomodulatory interventions in psoriasis treatment [18].

The central role of the IL-23 axis in psoriasis pathogenesis is supported by emerging evidence from experimental models, molecular studies, and the remarkable clinical efficacy of biologic agents targeting IL-17 and IL-23. However, although persistent activation of this pathway is believed to contribute to systemic inflammation, its direct involvement in carcinogenesis remains largely mechanistic and has not yet been conclusively demonstrated in human populations [11,12,18].

Inflammation and prothrombotic signaling are related through complex pathways that modulate endothelial integrity and systemic hemostasis. Cytokines like IL-1β, TNF-α, and IFN-γ differentially influence this prothrombotic endothelial response, as demonstrated by their variable capacity to stimulate thrombin generation and TF expression [14]. Specifically, the literature indicates that IL-1β serves as the most potent inducer of endothelial TF expression and thrombin generation, whereas TNF-α exhibits a milder but significant effect and, in contrast, IFN-γ exerts no direct inductive action on these specific mechanisms [14]. These findings highlight the importance of distinguishing cytokine-specific prothrombotic inflammatory profiles, as underlying cascades vary depending on the etiology of the disease [14]. Moreover, the prothrombotic impact of these cytokines depends on their temporal kinetics and systemic concentrations, explaining why clinical thrombotic episodes coincide with acute flares of chronic inflammatory diseases like psoriasis [20,21].

The role of inflammation in driving thrombogenesis is further amplified by platelet-activating factor (PAF), a potent phospholipid mediator that induces platelet aggregation and activates downstream signaling pathways favoring thrombosis [21]. PAF is synthesized by various immune and endothelial cells and has been implicated in the inflammatory pathogenesis of psoriasis. Its increased systemic presence may serve as a reliable biomarker for latent prothrombotic states [21]. Increased platelet activation in psoriatic patients enhances the pathogenic interaction between inflammation and thrombosis, reinforcing the progression of CVDs [21,22]. Ultimately, systemic inflammation mediated via the coordinated actions of cytokines and PAF establishes a highly prothrombotic microenvironment that increases the risk of adverse vascular events, particularly in patients suffering from chronic inflammatory diseases such as psoriasis [21].

Additionally, clinical evaluations of plasma galectin levels in psoriatic populations demonstrate a distinct correlation between psoriasis and accelerated atherosclerosis. High concentrations of galectin-1 (Gal-1) are strongly correlated with atherosclerotic plaque burden, and significantly elevated plasma levels of Gal-1 have been isolated from patients presenting with psoriasis [23]. Consequently, individuals with psoriasis exhibit a higher incidence of ischemic heart disease (IHD) and cerebrovascular events, an association that is further exacerbated by comorbid depression. Metabolic risk factors, including dyslipidemia, type II diabetes, hypertension, and obesity occur with greater frequency in these patients, while premature atherosclerosis and increased arterial stiffness further compound the total vascular risk [24]. Shared pathological mechanisms driving these conditions include systemic insulin resistance, chronic oxidative stress, and hypercoagulability, suggesting that targeted systemic anti-inflammatory interventions may provide substantial therapeutic utility in reducing CVD mortality [24].

Collectively, these findings indicate that chronic inflammation in psoriasis extends beyond cutaneous pathology and contributes to systemic vascular dysfunction through interconnected cytokine, platelet, and endothelial pathways. While substantial experimental and epidemiological evidence supports the contribution of these mechanisms to thrombo-inflammatory complications, their precise role in cancer initiation and progression remains an active area of investigation. Current evidence suggests a linkage between pro-tumorigenic microenvironments and sustained cytokine signaling, oxidative stress, and endothelial dysfunction, although further validation in large prospective clinical investigations is still needed [11,12,18,24].

### 3.1. Inflammatory Biomarkers in Psoriasis

Several markers of inflammation have been validated in patients with psoriasis, reflecting both clinical disease activity and underlying burden of systemic inflammation. These biomarkers encompass parameters derivable from routine complete blood counts (CBCs) as well as specialized molecular species involved in inflammatory cascades. Their routine clinical evaluation provides actionable insights for monitoring disease trajectories and assessing comorbidity risks. Specifically, statistically significant elevations in mean platelet volume (MPV) values, depressed serum concentrations of vitamin B_12_, and prolonged baseline coagulation parameters such as prothrombin time (PT), thrombin time (TT), and activated partial thromboplastin time (aPTT), have been observed more commonly in psoriatic cohorts compared to healthy controls [16].

The systemic immune–inflammation index (SII), calculated by the ratio of [(neutrophils × platelets)/lymphocytes], has emerged as a novel, high-impact biomarker reflecting localized immune responses and systemic inflammatory status [24]. When evaluated in psoriatic cohorts, elevated SII values show a positive correlation with baseline risk, with circulating monocyte and total white blood cell (WBC) counts acting as primary drivers of this statistical association [25]. Conversely, patients with psoriasis frequently exhibit lower absolute lymphocyte counts [25]. The relative risk of developing psoriasis increases as SII values increase. Further stratification demonstrates a significant positive correlation exclusively in females and a more pronounced risk profile within obese cohorts [24].

Investigating both the SII and the systemic inflammation response index (SIRI), researchers demonstrated that elevated baselines of both indices are positively associated with a higher risk of developing psoriasis [26]. Specifically, participants presenting moderate-to-high SII or SIRI values exhibited a significantly increased risk of psoriasis compared to low-index cohorts, a trend that remained statistically robust when the indices were evaluated as continuous variables [26]. Furthermore, assessments of classical inflammatory markers, such as the neutrophil-to-lymphocyte ratio (NLR), platelet-to-lymphocyte ratio (PLR), and human serum C-reactive protein (hsCRP), in chronic plaque psoriasis revealed that absolute neutrophil counts, hsCRP, and NLR were notably higher, whereas absolute lymphocyte counts were depressed. In some clinical subsets, however, variations in absolute platelet counts and PLR did not achieve statistical significance [27].

Comparative biochemical profiles between psoriatic cohorts and healthy controls demonstrated that psoriatic patients displayed markedly higher serum levels of hsCRP, serum amyloid A (SAA), erythrocyte sedimentation rate (ESR), WBCs, absolute neutrophils, NLR, and monocyte-to-high-density-lipoprotein ratio (MHR), alongside elevated aspartate aminotransferase (AST) markers, compared to healthy individuals. Conversely, high-density lipoprotein–cholesterol (HDL-C) levels were notably decreased in psoriatic patients compared to the control group. These distinct homeostatic deviations clearly reflect the pervasive systemic inflammatory state that characterizes the disease [28]. This is supported by separate clinical data demonstrating significant statistical variances in median hematocrit, total platelets, neutrophils, lymphocytes, red blood cell distribution width (RDW), ESR, CRP, and calculated SII values between healthy individuals and psoriatic cohorts, with all parameters except absolute lymphocyte counts being uniformly higher in the disease group [19]. Similarly, elevated CRP values consistently confirm a state of heightened systemic inflammation in the psoriatic population, even when raw platelet counts showed no significant deviation from healthy controls. Across multiple studies, psoriatic patients exhibited elevated hsCRP, ESR, NLR, SII, and SAA compared with healthy cohorts, supporting the presence of systemic inflammation [27].

Among the available indices, inflammatory biomarkers including NLR, PLR, SII, and hsCRP, although providing great clinical utility and valuable adjunctive information, should not be interpreted in isolation because they are influenced by several non-psoriasis-related factors (e.g., acute infections, obesity, metabolic syndrome, age, smoking status, and concomitant inflammatory disorders). As a result, they cannot replace established clinical measures such as the psoriasis area and severity index (PASI). Conversely, emerging biomarkers including SIRI, ESR, MHR, mean platelet volume (MVP), SAA, and RDW have exhibited promising associations with disease activity and systemic inflammation, but the available evidence remains largely inconclusive and less reproducible across different populations. Further large-scale prospective studies are therefore required before these biomarkers can be routinely integrated into clinical practice [27,29,30].

### 3.2. Disease Severity and Inflammation

Substantial research has focused on correlating the magnitude of these inflammatory biomarkers with clinical disease severity, quantified via the PASI index. For instance, data reveal a moderate negative correlation between PASI scores and the MPV index, coupled with a moderate negative correlation regarding vitamin B_12_ levels, while correlations with PT, TT, and aPTT indices did not reach statistical significance [16]. Moreover, disease severity was positively correlated with serum hsCRP, SAA, and MHR values, indicating that higher expressions of these inflammatory markers correspond to an advanced clinical phenotype [28]. In contrast, a negative correlation has been observed between PASI scores and LMR ratio, further validating the role of inflammatory dysregulation in driving disease pathophysiology [28].

Stratified analyses demonstrate that patients presenting with severe disease (PASI ≥ 4.5) are linked to significantly higher absolute WBCs, neutrophil counts, ESR, CRP, and calculated SII markers [19]. Furthermore, positive correlations have been confirmed between the PASI score, serum CRP levels, and the PLR ratio [27]. In a dedicated study, researchers observed markedly higher values for hematological inflammatory markers, including total WBC and neutrophil count, NLR, derived-NLR (d-NLR), SII, SIRI, and the aggregate index (AISI) as a function of advancing psoriasis severity together with a linear decline in absolute lymphocyte counts [31]. When testing the capacity of these markers to discriminate between distinct clinical stages, the NLR showed the highest diagnostic accuracy for predicting moderate psoriasis, whereas the d-NLR was identified as the sole biomarker significantly associated with severe forms of the disease [31]. Other standard metrics, such as raw neutrophil count, proved unreliable for differentiating severe from moderate clinical phenotypes [31]. Finally, a subset of epidemiological studies have reported no statistically significant correlation between select inflammatory markers and overall psoriasis severity, highlighting the heterogeneous nature of the disease [25,27].

Collectively, these findings indicate that the relationship between inflammatory biomarkers and psoriasis severity is more complex than a simple linear association. While NLR, SII, hsCRP, and several related indices correlate with increasing PASI scores, their strength and consistency vary due to differences in study design, sample size, patient demographics, disease duration, treatment status, prevalence of metabolic comorbidities (e.g., obesity and CVDs), laboratory methodologies, cut-off values, and statistical adjustment. Consequently, inflammatory biomarkers should be considered complementary rather than standalone indicators of disease severity, as their greatest clinical value may lie in combination with established clinical scoring systems like PASI [25,27].

### 3.3. Psoriasis Thrombo-Inflammatory Complications and Cardiovascular Diseases and Complications

Psoriasis is consistently associated with an increased burden of traditional CV risk factors and a higher prevalence of premature atherosclerosis. However, the elevated CVD risk observed in psoriatic patients is considered multifactorial, reflecting the combined effects of chronic systemic inflammation, obesity, dyslipidemia, metabolic syndrome, insulin resistance, atherosclerotic cardiovascular disease (ASCVD), ischemic stroke, smoking, genetic susceptibility, and other cardiovascular complications or lifestyle-related factors, rather than psoriasis alone [32,33,34,35]. This proatherogenic vascular phenotype is mediated, in part by platelet activation. Platelets represent core cellular mediators in endothelial dysfunction pathogenesis in CVD and represent primary therapeutic targets for secondary CVD prevention [35]. Specifically, platelets act as critical regulators of systemic inflammation, innate immune responses, and atherothrombosis through the intravascular release of numerous pro-inflammatory mediators [35].

Furthermore, due to the altered metabolic microenvironment of psoriatic skin, there are increased systemic levels of lipid and fatty acid mediators, including pro-inflammatory metabolites derived from both arachidonic and linoleic acid cascades. Transcriptomic analyses of isolated platelets from psoriatic patients have revealed overlapping pathways involved in CVD and active atherosclerosis, identifying a prominent gene expression signature centered on IFN signaling. The simultaneous presence of IFN-γ (type II) and IFN-α/β (type I) interferons, synthesized by discrete cell types within active psoriatic plaques, suggests that chronic exposure to these cytokines can structurally and functionally reprogram bone marrow megakaryocytes and circulating platelets, thereby amplifying systemic inflammation [35]. Persistent platelet activation may contribute to the enzymatic release of membrane-bound arachidonic acid, promoting the production of thromboxane A_2_ via the cyclooxygenase-1 (COX-1) pathway, which is related to vasoconstriction, microvascular injury, and increased CVD risk [35].

**Figure 3:**
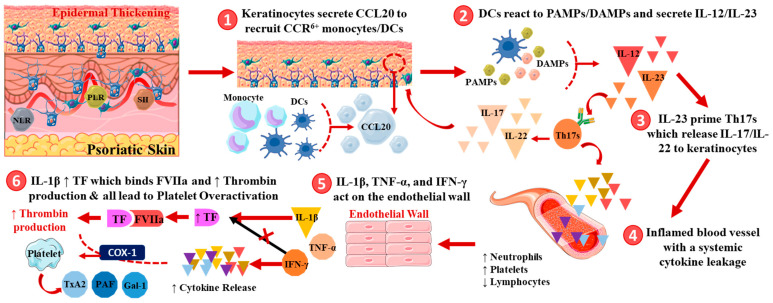
The cellular and molecular axis of psoriatic thrombo-inflammation and CVD risk. The step-by-step pathogenic sequence maps out: (**1**) cellular infiltration into psoriatic skin and localized secretion of chemokine CCL20; (**2**) antigen-presenting cells driving the upstream expression of IL-12 and IL-23 cytokines; (**3**) activation of the IL-12/IL-23 signaling inducing Th17 cell differentiation and subsequent production of IL-17 and IL-22; (**4**) systemic spillover of these pro-inflammatory cytokines into the vasculature; (**5**) cytokine-driven activation of the endothelial wall (via IL-1β, TNF-α, and IFN-γ), triggering increased TF expression; and (**6**) upregulation of the TF/VIIa complex, leading to increased thrombin production, COX-1 activation, and platelet-mediated release of pro-thrombotic factors (T_X_A_2_, PAF, and Gal-1) (parts of this figure were obtained from the free online databases https://smart.servier.com/ and https://www.flaticon.com/).

Collectively, these findings indicate that psoriasis is closely associated to an increased CVD risk but do not establish a direct causal relationship. Current evidence indicates that cardiovascular complications arise through a complex interaction between chronic system inflammation, genetic susceptibility, and traditional CVDs and lifestyle-related factors (e.g., obesity, smoking, and aging). Although inflammatory mediators such as TNF-α, IL-17, platelet activation, and thrombo-inflammatory signaling likely contribute to endothelial dysfunction and accelerated atherosclerosis, their relative contribution may differ substantially among individual patients [35]. The main mechanisms linking psoriasis-associated inflammation with CVDs are summarized in Figure 3.

## 4. Epidemiological Data on the Association of Psoriasis and Its Treatment with the Risk for Several Types of Cancer

### 4.1. The Association Between Psoriasis and Cancer in the General Population

A correlation between psoriasis and more than 23 different types of malignancies has been documented across the epidemiological literature. Associations between psoriasis and malignancies have been quantified using several epidemiological measures, including odds ratio (OR), relative risk (RR), hazard ratio (HR), and standardized incidence ratio (SIR). Because these measures capture different aspects of disease occurrence, they should not be interpreted interchangeably [36,37].

The reported oncological risk in patients with psoriasis varies according to the organ-specific type of cancer, alongside key patient characteristics including lifestyle factors, biological sex, and age [36,37]. Notably, the calculated risk estimates vary considerably between methodologies and specific cohorts, a divergence driven by variations in study design, statistical power, population demographics, and control parameters. These epidemiological findings should be interpreted alongside the biological mechanisms discussed in Section 3, where persistent IL-23/Th17-mediated inflammation, oxidative stress, endothelial dysfunction, and chronic immune activation provide plausible pathways linking psoriasis to carcinogenesis [36,37].

The following subsections summarize the available evidence according to individual malignancy type. Although each cancer is discussed separately, the overall interpretation should consider differences in study design, population characteristics, and analytical methodology, as these largely explain the observed variability across studies.

#### 4.1.1. Psoriasis and Colorectal Cancer (CRC)

The potential causal relationship between psoriasis and colorectal cancer (CRC), has been evaluated using advanced genetic frameworks. Bidirectional Mendelian randomization (MR) analyses suggested a small positive association between genetic susceptability to psoriasis and CRC (OR = 1.026, 95%, CI: 1.002–1.050, *p* = 0.037), although this finding was not consistently replicated across other MR datasets. This corresponds to a modest 2.6% increase in the odds of CRC in psoriatic patients compared to healthy individuals [2]. Conversely, a separate two-sample MR investigation yielded a negligible increase in CRC risk (OR = 1.0003) [7].

From an observational standpoint, a 10-year retrospective study conducted in the Dermatology Clinic of the “Sf. Spiridon” Emergency Hospital (Iasi, Romania), encompassing 1288 patients, evaluated whether psoriasis constitutes an independent risk factor of CRC. The authors reported a 28% increase in odds (OR = 1.28, 95%, CI: 0.48–3.46). However, this finding did not achieve statistical significance due to a wide confidence interval, suggesting that the variance could be driven by sample size limitations rather than a true causal relationship. Collectively, these findings highlight a lack of consensus regarding the potential impact of psoriasis on CRC risk [11].

#### 4.1.2. Psoriasis and Skin Cancer

The association between psoriasis and different classifications of skin cancer remains a subject of ongoing debate [7,11]. In a two-sample MR study, no statistically significant causal association was observed for general skin malignancies, with OR values remaining near unity (OR = 1.0002, 0.9999, and 1.0000), and all 95% confidence intervals including the null value of 1. Therefore, psoriasis does not appear to increase or decrease the risk of skin cancer [7]. Similarly, evaluations focusing on melanoma skin cancer (MSC) revealed no significant association, with calculated OR values hovering close to unity (OR = 0.9997, 0.9992, and 0.9994), and all 95% confidence intervals including the null value of 1.0 [7].

In contrast, a distinction emerges when evaluating non-melanoma skin cancer (N-MSC). A large-scale observational and genetic investigation reported a notable increase in terms of NMSC hazard, yielding an HR = 1.11 (95%, CI: 1.02–1.21). This indicates that individuals battling psoriasis exhibit an 11% increased likelihood of developing NMSC compared to the general population, an oncological predisposition mostly absent when evaluating MSC cohorts [11].

#### 4.1.3. Psoriasis and Breast Cancer (BC)

The epidemiological intersection between psoriasis and breast cancer (BC) has been documented across many studies [38]. For instance, a Phenome-Wide Association Study (PheWAS) demonstrated that patients with a genetic predisposition to psoriasis have marginal 1% higher odds of developing BC (OR = 1.01, 95%, CI: 1.00–1.01), with a greater predisposition observed in female patients [38]. To cross-validate these findings, the authors applied a one-sample MR analysis, which revealed a more pronounced 6% increase in the odds (OR = 1.06, 95%, CI: 1.01–1.12) [38]. Furthermore, a complementary two-sample MR analysis using the inverse-variance weighting (IVW) method showed a small but statistically significant 2% odds elevation (OR = 1.02, 95%, CI: 1.01–1.03) [38]. This methodological variation underscores how one-sample MR models can occasionally be prone to inflated effect estimates compared to two-sample architectures [38].

In a separate 10-year localized observational study, 42% higher odds of BC were found in psoriatic patients (OR = 1.42, 95%, CI: 0.47–4.26), though this observation lacked statistical significance due to the wide confidence interval [11]. Conversely, multi-cohort MR analyses evaluating separate datasets identified a slight 2.57% negative association within the Stuart dataset (OR = 0.9743, 95%, CI: 0.9484–1.0008), alongside a minor 1.4% positive association within the FinnGen dataset (OR = 1.0140, 95%, CI: 0.9974–1.0307), culminating in a pooled meta-analysis OR = 0.9954, indicating no overall causal effect [7]. Similarly, a bidirectional MR study exploring the relationship between AIDs and BC risk reported a 1.07% reduction in BC odds among psoriatic patients (OR = 0.9893, 95%, CI: 0.761–1.2846). Nevertheless, the generalizability of this specific finding is restricted by its exclusive reliance on European sample demographics [37].

Overall, the available evidence does not support a consistent causal association between psoriasis and BC. Although several genetic analyses reported statistically significant associations, the observed effect sizes were generally small and varied considerably between analytical approaches, indicating that any potential increase in risk is likely modest and should be interpreted cautiously [7,11,37,38].

#### 4.1.4. Psoriasis and Kidney Cancer (KC)

The potential link between psoriasis and kidney cancer (KC) was evaluated by performing statistical analyses PheWAS, polygenic risk score analyses, and one-sample and two-sample MR analyses. It was reported that psoriatic patients present 2% higher odds in developing KC (OR = 1.02, 95%, CI: 1.01–1.03). Concurrently, by applying a one-sample MR analysis it was revealed that the odds increased by 25% (OR = 1.25, 95%, CI: 1.09–1.42) [38]. When the less false-positive-prone two-sample MR analysis was applied to the same dataset, a 15% positive correlation was found, though it fell short of statistical significance (OR = 1.15, 95%, CI: 0.93–1.42) [38].

In contrast, a separate large-scale two-sample MR analysis revealed no causal association between psoriasis and KC risk across the Stuart dataset (OR = 1.0000), the FinnGen dataset (OR = 1.0001), or within their combined meta-analysis (OR = 1.0000) [7]. Taken together, the current evidence remains inconsistent. While some genetic analyses suggest a possible association, larger two-sample MR studies have not confirmed a causal relationship, indicating that additional large, prospective investigations are required [7,38].

#### 4.1.5. Psoriasis and Lung (LC) or Liver Cancer (LIC)

Investigating the relationship between psoriasis and various types of cancer, including lung cancer (LC), observational studies have suggested a modest increase in LC risk, reporting a 22% higher hazard of psoriatic patients developing the disease (HR = 1.22, 95%, CI: 1.01–1.48) [7,38]. Conversely, a two-sample MR analysis concluded that psoriasis does not causally influence LC risk, showing a negligible association across individual and pooled datasets (OR = 1.0003 (Stuart), OR = 1.0001 (FinnGen), and OR = 1.0003 (meta-analysis)) [7]. The same MR framework evaluated LIC susceptibility, confirming a total lack of association (OR = 0.9999 (Stuart) and 1.0001 (FinnGen)) [7].

However, a 10-year localized retrospective study encompassing 950 participants at the “Sf. Spyridon” Emergency Hospital Dermatology Clinic reported 161% higher odds of LC in psoriatic patients (OR = 2.61, 95%, CI: 0.71–9.68). This result was not statistically significant because the confidence interval crossed the null value of 1.0, an effect likely attributed to sample size limitations or regional confounding factors including smoking prevalence [11].

Overall, the available evidence favors a non-causal relationship between psoriasis and LC or LIC, with the positive associations reported in observational studies likely reflecting residual confounding by smoking, age, and other CVDs or metabolic risk factors rather than psoriasis itself [7,11,38].

#### 4.1.6. Psoriasis and Pancreatic Cancer (PC)

The potential causal association between psoriasis and pancreatic cancer (PC) has been investigated via two-sample MR analyses. Utilizing data from the Stuart, FinnGen, and integrated meta-analysis cohorts, researchers found no statistically significant association. The calculated OR values ranged from 0.9300 to 1.0482, with all corresponding 95% confidence intervals encompassing 1.0, indicating that any minor variations in observed PC risk are likely attributed to stochastic variation [7].

#### 4.1.7. Psoriasis and Central Nervous System Cancer (CNSC)

Data regarding central nervous system (CNS) malignancies show extreme divergence between study designs. A 10-year localized clinical registry study reported that individuals with psoriasis demonstrated 16.09 times higher odds of developing CNSC compared to healthy controls (OR = 16.09, 95%, CI: 2.61–99.4). While the confidence interval excludes 1.0, confirming a statistically robust local association, the extreme width of the interval indicates that this finding must be interpreted with caution, as it was likely inflated by a small absolute number of oncology cases within the study sample [11]. Conversely, a robust two-sample MR analysis utilizing larger genetic datasets revealed a complete absence of a causal link between psoriasis and brain malignancies, with OR values tightly centered around unity (OR = 1.0000 to 1.0001) and all confidence intervals including the null value of 1.0 in all cases [7].

#### 4.1.8. Psoriasis and Upper Digestive System (UDS) Cancer

The upper digestive system (UDS) involves the oral cavity (including the tongue, teeth, and salivary glands), pharynx, esophagus, stomach, and duodenum [39]. In a localized, 10-year analysis, psoriasis was strongly associated with increased odds of UDS malignancies, yielding an OR = 4.81 (95%, CI: 1.41–16.41). This implies that psoriatic patients carry an approximate 4.81-fold higher risk of developing UDS cancers compared to the general population. Although the confidence interval excludes the null value of 1.0, confirming statistical significance, its wide range introduces numerical uncertainty regarding the exact magnitude of the risk, implying a critical need for larger multi-center studies [7].

When evaluating specific organs within the UDS, findings vary. Whether psoriasis is causally associated with gastric cancer (GC) has been investigated using MR analysis. These analyses suggested a nominal, non-significant 0.37% odds reduction (OR = 0.9963, 95%, CI: 0.9724–1.0207, *p* = 0.7622) [1]. Conversely, a large-scale systematic review and meta-analysis of a pooled cohort encompassing over half a million patients identified a statistically significant positive association, revealing that the standardized incidence of GC was 19% higher than expected (SIR = 1.19, 95%, CI: 1.04–1.36, P_heterogeneity_ = 0.59). The low heterogeneity across the included studies underscores the stability and reliability of this positive correlation [40]. In contrast, esophageal cancer risk evaluated via a combined meta-analysis of the Stuart and FinnGen datasets showed complete absence of a causal nexus (OR = 1.0001 (95%, CI: 0.9997–1.0003)) [7].

Collectively, the available evidence suggests that associations within UDS tract malignancies differ substantially by cancer subtype. While observational evidence supports an increased incidence of gastric cancer, genetic studies have not consistently demonstrated causality, highlighting the complexity of interpreting these associations and the need for larger prospective investigations [7,38].

#### 4.1.9. Psoriasis and Mesothelioma

Malignant mesothelioma is an aggressive type of cancer originating within the thin mesothelial tissue lining the lungs, chest wall, and abdominal cavity [41]. An epidemiological evaluation combining observational and genetic datasets reported a borderline significant 54% elevated hazard of mesothelioma among psoriatic cohorts (HR = 1.54, 95%, CI: 1.00–2.37). However, because the 95% confidence interval touches the null value of 1.0, a potential role of random chance or external factors like occupational asbestos exposure, cannot be definitively excluded [38].

#### 4.1.10. Psoriasis and Bladder Cancer (BLC)

The potential causal association between psoriasis and bladder cancer (BLC) has been evaluated using two-sample MR models. A lack of association was suggested by an independent two-sample MR analysis across separate datasets, with calculated OR values ranging tightly from 1.0000 to 1.0001 [7]. In contrast, a localized retrospective clinical study reported that the risk of BC was 3.15 times higher in individuals presenting with psoriasis (OR = 3.15, 95%, CI: 0.83–11.97) [11]. However, this finding lacks statistical significance due to a wide confidence interval overlapping the null value of 1.0, indicating substantial uncertainty of the true magnitude of this association in smaller cohorts [11].

#### 4.1.11. Psoriasis and Non-Hodgkin’s Follicular Lymphoma (NHL)

Psoriasis exhibits a distinct association with the development of non-Hodgkin’s follicular lymphoma (NHL), an effect validated by two separate statistical approaches. Specifically, utilizing a one-sample MR framework, researchers identified a significant 32% increase in the odds of developing this hematological malignancy (OR = 1.32, 95%, CI: 1.07–1.64) [38]. Concurrently, a PheWAS model tracking genetic predisposition via a calculated polygenic risk score (PRS) demonstrated a weaker but still statistically significant positive association (OR = 1.02, 95%, CI: 1.00–1.04). Together, these complementary genetic analyses support a potential causal association of psoriasis to follicular NHL, even though the absolute size varies between analytical models [38].

Among the malignancies evaluated, follicular NHL represents one of the more consistently supported positive associations across different genetic analytical approaches. Nevertheless, additional validation in independent populations remains necessary before definitive causal conclusions can be drawn [38].

#### 4.1.12. Psoriasis and Endocrine Cancer (EC)

The epidemiological intersection between psoriasis and malignancies of the endocrine system was evaluated in a 10-year hospital registry cohort. The reported data suggested a potential 210% odds increase among psoriatic patients (OR = 3.10, 95%, CI: 0.8–11.97). However, this result was not statistically significant, as the confidence interval includes 1.0 and the wide distribution (0.8–11.97) indicates substantial numerical uncertainty, necessitating larger pooled datasets to confirm or refute the trend [11].

#### 4.1.13. Psoriasis and Secondary Malignant Neoplasm of Other Sites (SOS)

The risk of developing a secondary malignant neoplasm of other sites (SOS) has been evaluated through parallel genetic analyses. A baseline PheWAS model revealed a small but statistically significant elevated risk among psoriatic patients, reporting an (OR = 1.01, 95%, CI: 1.00–1.01). When a one-sample MR analysis was executed on the same population, it demonstrated a more pronounced 10% increase in the odds (OR = 1.10, 95%, CI: 1.03–1.17). The statistical robustness of this finding supports the hypothesis that psoriasis may be causally linked to an increased risk of SOS [38].

#### 4.1.14. Psoriasis and Other Cancers Without Causal Association

Beyond the malignancies detailed above, large-scale epidemiological screening has successfully ruled out causal links for several other cancer types. Specifically, a comprehensive two-sample MR analysis pooling data across multiple cohorts confirmed that there is no statistically significant evidence linking a diagnosis of psoriasis to an increased susceptibility for head and neck cancers, multiple myeloma, or biliary tract malignancies [7]. The epidemiological risk indicators and statistical boundaries for these various tissue-specific malignancies are systematically summarized in Table 1.

Overall, the epidemiological evidence demonstrates substantial heterogeneity regarding the association between psoriasis and individual cancer types. While relatively consistent positive associations were observed for NMSC, follicular NHL, and SOS, the evidence for several malignancies including CRC, BC, KC, LC, gastric, BLC, and CNSC, remains conflicting across different analytical approaches. In contrast, MR analyses consistently found no convincing evidence supporting a causal association between psoriasis and melanoma, LIC, PC, and esophageal cancers. Cancer type and differences in study design, sample size, and confounding factors (e.g., obesity and smoking), determine the strength of the consistency of evidence [7,30,42].

Interpretation of the available epidemiological evidence should consider several important confounding factors, as many malignancies discussed in this review are strongly influenced by cancer risk determinants including smoking, obesity, alcohol consumption, metabolic syndrome, aging, occupational exposures, and genetic susceptibility. All these factors frequently co-exist in patients with psoriasis and may partially explain the increased cancer incidence reported in some observational studies. Disease severity, treatment history, cumulative drug exposure, and divergent healthcare utilization may additionally influence the reported associations [7,30,42].

Consequently, statistical significance should not automatically be interpreted as clinical or biological significance, particularly when reported effect sizes remain close to unity. Several statistically significant associations identified in genetic or large population-based studies correspond to relatively small increases in relative risk, and therefore should be interpreted cautiously within the broader context of biological plausibility, study design, and potential confounding factors. Moreover, the true relationship between psoriasis and malignancy is likely multifactorial and should be interpreted within the broader clinical context rather than as direct causal association [7,30,42].

### 4.2. Association of Psoriasis and Cancer in a Specific Population Group

Beyond general population assessments, epidemiological research has stratified the oncological risks associated with psoriasis across specific demographics defined by biological sex and age. Delineating these risk profiles is essential, as the intersections of chronic inflammation with sex-specific endocrine backgrounds and age-related immunosenescence can modulate carcinogenesis. The statistical associations between psoriasis and tissue-specific malignancies in female, male, and geriatric populations (aged 65 years and older) are discussed below.

While Section 4.1 focuses on the baseline association between psoriasis and cancer, the following section examines whether therapeutic interventions modify this risk. Importantly, treatment-related risk should be interpreted separately from disease-related risk because chronic inflammation, disease severity, cumulative drug exposure, and patient characteristics may all influence oncological outcomes.

#### 4.2.1. Psoriasis and Cancer in the Female Population

The epidemiological relationship between AIDs and female-specific malignancies, particularly BC, has been extensively evaluated. Utilizing an IVW random-effects model, an aggregate analysis demonstrated a modest but significant 9% increase in BC relative risk among psoriatic patients (RR = 1.09, 95%, CI: 1.03–1.15) [37]. Subsequent sensitivity and leave-one-out analyses verified the robustness of the findings. Notably, upon the exclusion of an influential study by Stern et al. [37], the positive relationship remained robust, exhibiting an updated RR of 1.08 (95%, CI: 1.04–1.12). However, accompanying meta-analyses failed to demonstrate a statistically significant association due to the confidence intervals including the null value. These discrepancies underscore potential population-specific influences, highlighted by marked geographic heterogeneity across Asian, European, South American, and Australian cohorts [37].

Concurrently, multi-method investigations combining observational data and MR frameworks support an elevated risk profile in female cohorts. An observational PheWAS study identified a statistically significant increase of BC hazard among psoriatic women, yielding an HR of 1.24 (95%, CI: 1.11–1.38). When evaluated via an MR design to estimate genetic causality, a milder but still statistically significant positive association was confirmed (OR of 1.06, 95%, CI: 1.02–1.11) [38]. Overall, current evidence suggests that psoriatic women may have a modestly increased risk of BC, although the magnitude of the association varies across study designs. Risk indices range from 1.06 to 1.24 depending on the statistical framework and sample demographics utilized [37,38].

Regarding other female-specific gynecological malignancies, large-scale genetic data do not support a causal association between psoriasis and either cervical cancer (meta-analysis: OR = 1.0000, 95%, CI: 0.9996–1.0004) or ovarian cancer [7]. Nevertheless, when screening non-sex-specific malignancies within female-only cohorts, several elevated risks have been reported. These include anal canal carcinoma (OR = 1.61, 95%, CI: 1.12–2.32), NMSC (OR = 1.07, 95%, CI: 1.01–1.14), and follicular NHL, where the risk appears elevated by approximately 50%. Additionally, women diagnosed with psoriasis exhibit a modest, statistically significant increase in the likelihood of developing SOS (OR = 1.12) [38].

#### 4.2.2. Psoriasis and Cancer in the Male Population

Oncological susceptibilities in male populations with genetic predispositions to psoriasis have been characterized via comparative PheWAS and MR analyses. Delineating these associations reveals a consistent trend toward elevated risks for respiratory, renal, and gastrointestinal malignancies, though the calculated magnitude of the odds varies among statistical models [38]. For LC, a baseline PheWAS analysis demonstrated 1% higher odds, whereas the corresponding MR analysis indicated a 17% higher genetically predicted risk. Similarly, the calculated odds for KC rose by 2% under the PheWAS model but escalated to a 34% increase under the MR framework. Finally, systemic rheumatic diseases (SRDs) exhibited a 1% odds elevation in the initial PheWAS analysis, compared to a 10% odds increase identified via MR analysis [38].

Complementary observational PheWAS data tracking men with clinically diagnosed psoriasis identified distinct organ-specific hazards. Psoriatic males demonstrated a statistically significant elevation in LC hazard, reporting an HR of 1.22 (95%, CI: 1.01–2.14). Furthermore, male-specific urological and oral malignancies were strongly represented: the hazard for penile cancer was elevated 3.02-fold (HR = 3.02, 95%, CI: 1.45–6.29), BLC hazard increased by 23% (HR = 1.23, 95%, CI: 1.00–1.51), and oral cavity carcinoma hazard rose by 294% (HR = 2.94, 95%, CI: 1.05–8.27) [38]. While the confidence intervals for both penile and oral malignancies are wide, they exclude the null value, indicating statistical significance and an expected degree of numerical uncertainty [38].

In contrast, findings regarding prostate cancer susceptibility remain highly polarized across the literature [7,11]. A large-scale pooled meta-analysis identified a small but statistically significant negative association, reporting 0.19% slightly lower odds of prostate cancer development among psoriatic males (OR = 0.9981, 95%, CI: 0.9969–0.9994) [7]. Conversely, a localized hospital registry study reported a 46% increase in risk (OR = 1.46, 95%, CI: 0.42–5.12) [11]. The extreme width of this latter confidence interval, paired with its reliance on a small sample size from a single geographic region, suggests that this positive association should be interpreted with caution and may be driven by localized confounding factors or screening biases [1,11].

#### 4.2.3. Psoriasis and Cancer in Individuals Aged ≥66 Years

The clinical intersections between AIDs and malignancy have also been evaluated in geriatric populations. A large-scale epidemiological study focusing on a United States (US) cohort of individuals aged 66 years and older investigated the baseline odds of developing anal carcinoma. For the psoriatic subgroup within this elderly population, initial statistical modeling demonstrated a notable positive association, reporting a 28% increase in anal cancer odds (OR = 1.28, 95%, CI: 1.06–1.56). However, due to multiple-testing adjustments across the wider autoimmune dataset, this finding did not retain statistical significance after the application of a stringent Bonferroni correction [36].

When the geriatric psoriatic cohort was stratified by biological sex, sex-specific differences emerged between elderly men and women. In older males presenting with psoriasis, the odds ratio for anal carcinoma was markedly higher and remained statistically significant, yielding an OR of 1.68 (95%, CI: 1.03–4.28). In contrast, the association among elderly women with psoriasis was weaker and failed to reach statistical significance (OR = 1.12, 95%, CI: 0.88–1.43) [36].

Epidemiological evidence links psoriasis to increased risk across several malignancies, though association strength and consistency vary depending on study design and the population examined. Large observational cohorts provide valuable real-world data but remain susceptible to residual confounding from disease severity, smoking, alcohol consumption, obesity, and treatment exposure. While meta-analyses enhance statistical power and risk estimate precision, they are inherently limited by baseline study heterogeneity. Conversely, MR studies minimize reverse causation and environmental confounding to strengthen causal inference, yet fail to fully capture the complex gene–environment interactions that characterize psoriasis.

Discrepancies between observational and genetic data reflect the distinct strengths and limitations of these complementary epidemiological approaches rather than outright contradictions. Notably, several reported associations rely on few cancer cases, yielding wider confidence intervals and low statistical precision, while differences in patient demographics, geography, and healthcare systems drive further heterogeneity. Currently, NMSC and certain lymphomas represent the most robust epidemiological associations. In contrast, evidence linking psoriasis with several solid tumors remains inconsistent and understudied in larger cohorts. Ultimately, accurately evaluating cancer risk in psoriasis requires integrating these diverse, complementary study designs rather than relying on any single epidemiological approach.

## 5. Epidemiological Results for Patients with Psoriasis Under Treatment: Association with Cancer

Treatment options for psoriasis vary depending on disease severity [6]. Therapeutic modalities are broadly classified into topical, conventional systemic, and biologic interventions. Topical therapies, such as corticosteroids and vitamin D_3_ analogues, are standard for mild manifestations [6]. Conversely, moderate-to-severe plaque psoriasis necessitates systemic regimens, utilizing either conventional synthetic disease-modifying antirheumatic drugs (csDMARDs) or targeted biological agents designed to modulate underlying immune dysregulation [6,43]. Both conventional systemic therapies and biologic agents achieve therapeutic efficacy primarily by mitigating chronic, systemic inflammatory pathways [44]. In standard clinical algorithms, conventional systemic agents serve as first-line treatment options for patients requiring non-topical interventions. If these conventional therapies induce iatrogenic toxicity, exhibit poor tolerability, or fail to achieve adequate disease control, escalation to biological therapies is indicated [45].

Historically, modalities such as ultraviolet phototherapy and non-biological systemic agents including acitretin, cyclosporine, and methotrexate formed the backbone of moderate-to-severe psoriasis management. However, elucidation of the immunopathophysiology of the disease has catalyzed the development of targeted biological therapeutics. These include TNF-α inhibitors, IL12/23 and IL-17 inhibitors, which drive the downstream inflammatory feedback loop and accelerate keratinocyte hyperproliferation. Because such cytokines are central drivers of psoriasis pathogenesis, they have become major therapeutic targets for the management of moderate-to-severe disease. While biological therapies target these precise cytokines or their receptors to achieve clinical remission, primary or secondary therapeutic resistance may occur, causing some biological agents to lose their therapeutic efficacy over time [46].

Cytokines including TNF-α, IL-12, IL-23 and IL-17 are essential not only for executing inflammatory responses but also for maintaining physiological immune surveillance and preventing oncogenesis [47]. Systematic reviews indicate that methotrexate, cyclosporine, and TNF-α inhibitors are generally considered safe options for psoriasis management [48]. Nonetheless, conflicting evidence suggests that specific long-term exposures, including psoralen plus ultraviolet A (PUV_A_), ultraviolet B (UV_B_), methotrexate (MTX), cyclosporine (CsA), mycophenolate mofetil (MMF), and various biological agents, may be associated with an increased risk of treatment-related malignancies [49].

### 5.1. Non-Biological Treatments and Cancer Risk

#### 5.1.1. Phototherapy

Phototherapy utilizing ultraviolet (UV) radiation is an established treatment option for moderate-to-severe psoriasis. UV radiation downregulates cutaneous dendritic cell activity and suppresses the activation of pathogenic effector T cells, thereby reducing local inflammation. However, its long-term deployment carries risks regarding skin cancer.

Clinical data demonstrate that PUV_A_ may increase the incidence of skin malignancies dose dependently due to its concurrent immunosuppressive and mutagenic effects, though it does not appear to alter the baseline risk for internal malignancies [49]. Conversely, broad-band and narrow-band (NB) UV_B_ therapies show no statistically significant causal association with internal or cutaneous malignancies [47]. At the molecular level, UV radiation can induce direct DNA photoproducts within epidermal keratinocytes, resulting in DNA mutations that drive carcinogenesis if immune repair mechanisms are saturated [6]. Epidemiological evaluations of cancer risk in psoriatic cohorts undergoing PUV_A_ and UV_B_ phototherapy have focused heavily on NMSC, encompassing squamous cell carcinoma (SCC) and basal cell carcinoma (BCC), alongside cutaneous malignant melanoma. Longitudinal surveillance reveals that the relative risk of developing NMSC increases following more than 35 cumulative UV_B_ sessions or more than 10 PUV_A_ exposures. More specifically, the hazard for both BCC and SCC escalated markedly after exceeding 100 total PUV_A_ sessions, whereas an increased risk for melanoma emerges mainly in patients exposed to more than 200 PUV_A_ sessions [50].

In a large-scale cohort study investigating malignancy risk across distinct treatment modalities over a 12-month minimum exposure period, phototherapy yielded an HR of 1.13 for overall malignancies (excluding NMSC), an HR of 1.39 for general skin cancers, and an HR of 1.45 specifically for NMSC. Despite these elevated point estimates, the authors concluded that phototherapy did not generate a statistically significant increase in the absolute risk of overall oncogenesis or NMSC within the evaluated cohort [47]. Furthermore, recent comparative data evaluating BCC incidence post-phototherapy confirmed a positive association with PUV_A_ regimens, whereas no similar oncological risk was identified for NB-UV_B_. This reinforces the clinical view that NB-UV_B_ represents a safer long-term phototherapeutic profile, though the small absolute size of some PUV_A_ treatment cohorts requires cautious interpretation of these comparative safety margins [49].

#### 5.1.2. Conventional Systemic Non-Biological Therapies

##### Methotrexate

Methotrexate (MTX) is a folic acid antagonist possessing potent immunosuppressive and anti-inflammatory properties that have been widely used in the treatment of psoriasis and psoriatic arthritis for over seven decades [51]. MTX acts mainly through the inhibition of dihydrofolate reductase (DHFR), thereby disrupting purine synthesis and halting the proliferation of rapidly dividing immune cells. Given this broad antimetabolite mechanism of action, its potential association with systemic and cutaneous oncogenesis has been extensively scrutinized [47].

Current evidence across multiple studies supports the relative safety of MTX regarding overall cancer risk in patients with psoriasis. Longitudinal tracking indicates that the absolute risk of overall malignancy is not elevated in patients receiving MTX, regardless of cumulative treatment duration when compared to non-exposed psoriatic controls [48]. Concurrently, large-scale registry data confirmed that MTX exposure was not associated with a significant risk elevation for overall malignancies, excluding NMSC, yielding an HR of 0.92. Moreover, no statistically significant variances were identified for solid organ or hematological malignancies between MTX-treated patients and non-psoriatic participants [47].

Supplementary data confirm this favorable oncological safety profile. MTX therapy does not demonstrate a significant association with the development of cutaneous SCC, and while a marginal elevation in BCC risk has been suggested, it remains epidemiologically unconfirmed [51]. Similarly, multi-center evaluations found no significant association between MTX use and aggregate neoplasia risks in patients exposed to conventional antirheumatic drugs (csDMARDs) [49,52].

A dedicated skin cancer registry analysis reported a statistically significant increase in BCC risk (OR = 1.43, 95%, CI: 1.23–1.67), whereas parallel risks for cutaneous SCC (OR = 1.18, 95%, CI: 0.80–1.74), and melanoma (OR = 1.15, 95%, CI: 0.77–1.72) were not statistically significant [53]. In summary, while MTX does not increase the overall systemic malignancy burden, a potential role in facilitating specific low-grade cutaneous malignancies like BCC, cannot be entirely discounted.

##### Cyclosporine

Cyclosporine (CsA) is a potent calcineurin inhibitor approved for the treatment of severe recalcitrant psoriasis. At the molecular level, it binds to the intracellular cyclophilin receptor, forming a cyclosporin–cyclophilin complex that inhibits the phosphatase activity of calcineurin. This lock prevents the dephosphorylation and nuclear translocation of the nuclear factor of activated T-cells (NFAT), selectively suppressing T-cell activation and IL-2 transcription. However, prolonged CsA-induced systemic immunosuppression compromises physiological tumor surveillance, increasing the risk of malignancies, particularly lymphoproliferative disorders and NMSCs [47].

Epidemiological data confirm that CsA exhibits a less favorable oncological safety profile compared to other conventional systemic options. Long-term CsA therapy drives a notable increase in NMSC incidence, specifically favoring cutaneous SCC, with a reported RR of 3.3. Furthermore, the overall risk of malignancy escalates during extended CsA deployment (OR = 1.57), whereas short-term, intermittent regimens do not demonstrate a statistically significant increase in cancer risk [48]. Complementary cohort evaluations confirm this heightened risk profile, showing that CsA exposure yields an adjusted hazard ratio (aHR) of 1.20 (95%, CI: 1.04–1.39) for overall malignancies excluding NMSC. When looking at organ-specific systems, CsA significantly increases pancreatic adenocarcinoma hazard (HR = 1.97, 95%, CI: 1.25–3.10) and hematological malignancies, particularly NHL (HR = 3.46, 95%, CI: 1.90–6.30) [47]. These findings suggest that CsA is associated with an increased risk of both cutaneous and visceral malignancies. Moreover, clinical evidence indicates that combining CsA with PUV_A_ phototherapy further compounds this carcinogenic risk, though sample size limitations currently prevent a precise numerical determination of this synergistic toxicity [50]. Ultimately, long-term CsA usage supports regular oncological surveillance, distinguishing it from more favorable non-biological systemic alternatives.

##### Acitretin

Acitretin is a second-generation oral synthetic retinoid that acts by binding to nuclear retinoic acid receptors, thereby normalizing epidermal cell proliferation, downregulating keratinocyte hyperproliferation, and modulating structural differentiation. Beyond its anti-psoriatic efficacy, acitretin possesses distinct antineoplastic and chemopreventive properties, and is routinely used to suppress skin cancer development in immunosuppressed organ transplant recipients. Additionally, when deployed as a combination strategy alongside UV phototherapy (Re-UV_B_ or Re-PUV_A_), acitretin can mitigate the skin cancer risks associated with phototherapeutic radiation [47].

Longitudinal evaluations tracking psoriatic patients exposed to acitretin for a minimum of 12 months confirmed no significant increase in the risk of total malignancies, excluding NMSC, reporting an HR of 1.03. Moreover, no statistically significant elevation for any distinct malignancy subtype was observed in the acitretin group compared to non-psoriatic control populations [47]. Independent retrospective evaluations also confirmed the absence of a statistically significant association between acitretin treatment and increased cancer risk [49]. Consequently, acitretin represents an oncologically favorable systemic option, though continued long-term observational studies remain necessary to fully define its extended safety boundaries.

##### Sulfasalazine

Sulfasalazine is a conjugate of 5-aminosalicylic acid and sulphapyridine that is less frequently deployed in contemporary psoriasis management compared to alternative conventional systemic therapies. Available epidemiological data indicate no statistically significant variance in the overall risk of malignancy among psoriatic patients receiving sulfasalazine for over 12 months when compared to systemic-naïve psoriatic controls [48]. Nevertheless, because the total volume of clinical data for sulfasalazine in psoriasis patients remains limited, further extensive investigations are required to formulate definitive long-term safety conclusions.

#### 5.1.3. Targeted Synthetic Therapies: JAK Inhibitors

Targeted therapies for immune-mediated diseases include monoclonal antibodies directed against cytokines or their receptors, small molecule Janus kinase inhibitors (JAKis), and emerging JAK-signal transducer and activator of transcription (STAT) inhibitors. JAKis represent a class of targeted synthetic small-molecule therapies that reversibly interfere with the intracellular STAT signaling cascade. By binding to the adenosine triphosphate (ATP)-binding pockets of specific JAK enzymes (JAK1, JAK2, JAK3, and tyrosine kinase 2 (TYK2)), these small molecules block downstream signal transduction for multiple pro-inflammatory cytokines. Contemporary targeted synthetic therapies are broadly categorized into three clinical drug subtypes: anti-cytokine/receptor monoclonal antibodies, small molecule JAK inhibitors, and targeted STAT inhibitors [54].

Several JAKis, including tofacitinib, baricitinib, upadacitinib, and deucravacitinib, have demonstrated efficacy in mitigating immune-mediated diseases. Tofacitinib was the first Janus kinase inhibitor (JAKi) approved for inflammatory arthropathies such as rheumatoid arthritis (RA), followed by the development and approval of baricitinib, upadacitinib, filgotinib, peficitinib [55], ruxolitinib [56], and abrocitinib, all of which demonstrate substantial efficacy in reducing clinical symptoms in moderate-to-severe psoriasis [54]. Concurrently, deucravacitinib (DEU) has been introduced as a highly selective, first-in-class allosteric inhibitor targeting the pseudokinase domain of TYK2, specifically designed to minimize off-target JAK1-3 signaling [57]. The clinical indications for this drug class have expanded to encompass psoriatic arthritis, plaque psoriasis, axial spondylarthritis, inflammatory bowel disease (IBD), and atopic dermatitis (AD) [55].

Given that the JAK-STAT pathway regulates hematopoiesis and immune cell development, its sustained pharmacological inhibition has raised questions regarding downstream immune surveillance and iatrogenic oncogenesis. In the contemporary psoriasis literature, the malignancy risk of JAKis is primarily evaluated through active-comparator trials, matching them against traditional TNF-α inhibitors and MTX.

### 5.2. Topical Treatments

Topical treatments are the first-line treatment for the management of mild psoriasis. These therapies encompass topical corticosteroids, vitamin D_3_ analogues, calcineurin inhibitors, keratolytic agents and certain forms of phototherapy [4,58].

Topical corticosteroids remain the most frequently prescribed intervention for patients presenting with limited plaque distribution. They diffuse across cell membranes to bind glucocorticoid receptors, reducing the transcription of pro-inflammatory genes and upregulating anti-inflammatory proteins. This signaling cascade suppresses downstream cytokine pathways, leukocyte infiltration, epidermal hyperproliferation, vascular permeability and vasodilation. The clinical selection of corticosteroid potency is dictated by the specific anatomical site to minimize iatrogenic local toxicities such as cutaneous atrophy. Fixed-dose combination formulations, such as a topical corticosteroid paired with a vitamin D_3_ analogue or keratolytic agents (e.g., tazarotene), enhance therapeutic efficacy while reducing individual adverse event profiles compared to monotherapy [4,58].

Topical vitamin D_3_ analogues (e.g., calcipotriol) bind to nuclear vitamin D receptors, where they exert anti-proliferative actions on epidermal keratinocytes and promote cellular differentiation. While these agents can induce transient irritation or a burning sensation upon initial application, these adverse symptoms typically resolve with continued treatment. Topical calcineurin inhibitors (TCIs), such as tacrolimus and pimecrolimus, are mainly reserved for thin, highly sensitive skin areas, including facial, intertriginous, and flexural regions. TCIs block the dephosphorylation of NFAT, suppressing T-cell activation and downregulating the production of inflammatory cytokine, such as IL-2 and IFN-γ, and are generally associated with mild local adverse effects [4,58].

Keratolytic agents, including tazarotene and salicylic acid, are deployed to desquamate and break down the thick hyperkeratotic scales covering psoriatic plaques. Tazarotene, a topical third-generation retinoid, modulates gene expression to normalize cell proliferation and reduce epidermal thickening, whereas salicylic acid reduces intercellular adhesion between keratinocytes, thereby promoting exfoliation and enhancing the penetration of co-administered topical agents. Finally, targeted local phototherapy, such as the 308 nm excimer laser or light therapy, utilizes monochromatic ultraviolet wavelengths to treat recalcitrant, localized psoriatic plaques. The likelihood of carcinogenesis associated with targeted modalities is low and substantial clinical improvement is frequently documented within two months of treatment initiation [58].

Within the scope of the present literature review, no epidemiological studies specifically evaluating long-term cancer risk in patients receiving exclusively topical treatments were identified. This gap in the literature exists because most longitudinal oncology registries rely on moderate-to-severe psoriatic cohorts exposed to chronic, systemic immunomodulatory interventions [4,58].

### 5.3. Biological Therapies and Cancer Risk

#### 5.3.1. Inhibitors of TNF-α

TNF-α inhibitors constitute a pivotal class of targeted biological agents designed to suppress TNF-α, a key pro-inflammatory cytokine central to the pathogenesis of systemic inflammation and psoriatic plaques. Prominent therapeutic agents within this classification include etanercept, infliximab, and adalimumab. These biologics exhibit high clinical efficacy in the long-term management of moderate-to-severe psoriasis, typically achieving high rates of skin clearance within 12 to 16 weeks of treatment initiation. A notable exception is infliximab, which demonstrates a more rapid onset of action, with clinical responses observed by weeks 8 to 10. However, the sustained neutralization of systemic TNF-α is associated with severe class-specific adverse effects. These include a heightened susceptibility to opportunistic infections, reactivation of latent viral hepatitis B and C (HBV and HCV), reactivation of latent tuberculosis (TB), drug-induced lupus erythematosus (DILE), and demyelinating disorders of the CNS [58].

Furthermore, because TNF-α plays an essential role in physiological immune surveillance and apoptosis, its chronic pharmacological inhibition has raised concerns regarding carcinogenesis. Epidemiological data evaluating psoriatic populations exposed to TNF-α antagonists have yielded variable results. A large cohort study reported an HR of 1.19 for overall systemic malignancies, excluding NMSC [47]. More specifically, a nationwide retrospective study found an elevated risk for hematological and cutaneous malignancies among South Korean psoriatic patients managed with biological therapies. The authors noted an aHR of 2.93 (95%, CI: 1.01–8.50) for total lymphoma, an aHR of 2.98 (95%, CI: 1.02–8.69) for NHL, and an aHR of 3.46 (95%, CI: 1.07–11.21) for primary cutaneous lymphoma [59].

These findings correlate with those of a parallel national cohort registry intervention comparing biological to non-biological interventions. While TNF-α inhibitors did not show a statistically notable correlation with overall myeloid or broad hematologic malignancies, they were associated with an increased risk of lymphoid-specific oncogenesis. The study reported an incidence rate ratio (IRR) of 1.78 (95%, CI: 1.08–2.93) for lymphoid malignancies, an IRR of 2.78 (95%, CI: 1.50–5.18) for general lymphoma subsets, and an IRR of 2.48 (95%, CI: 1.28–4.80) for NHL [60]. Conversely, a companion evaluation by Kim et al. [59] demonstrated only a marginal, non-significant increase in the aggregate risk of developing systemic malignancies [59]. Taken together, these epidemiological data suggest that while evidence for an elevated overall cancer risk remains inconsistent, long-term exposure to TNF-α inhibitors may be associated with an increased risk for specific lymphoid malignancies [61].

#### 5.3.2. IL-12/23 Inhibitors

The therapeutic targeting of the IL-23 signaling pathway represents a highly effective intervention for moderate-to-severe psoriasis. This pharmacological class includes ustekinumab, guselkumab, risankizumab, tildrakizumab, and mirikizumab, the latter of which is undergoing late-stage clinical development. Ustekinumab stands as the foundational biological agent in this category, it is a fully human monoclonal antibody that targets the shared *p40* subunit of IL-12 and IL-23 cytokines, thereby blocking their interaction with the IL-12R/β1 receptor complex [58]. In contrast, guselkumab is a fully human monoclonal antibody that binds and selectively inhibits the *p19* subunit of IL-23, thereby limiting inflammatory responses involved in psoriasis pathogenesis [62].

Long-term pharmacovigilance and registry data on guselkumab suggest a highly favorable safety profile. A 5-year longitudinal safety evaluation pooling data from clinical trials of guselkumab reported a low overall incidence rate (IR) for NMSC, quantified at 0.34 events per 100 patient years (PYs) of exposure [62]. Similarly low incidence rates were documented for solid organ and hematological malignancies, including BC (IR: 0.08/100 PY), CRC (IR: 0.07/100 PY), cutaneous malignant melanoma (IR: 0.06/100 PY), prostate carcinoma (IR: 0.06/100 PY), systemic lymphoma (IR: 0.03/100 PY), and SCC of the head and neck cancer (IR: 0.04/100 PY) [62]. Ultimately, the authors concluded that selective IL-23 inhibition via guselkumab exhibits a long-term malignancy risk profile comparable to established biological therapies targeting the IL-17 (secukinumab, ixekizumab) or IL-12/23 (ustekinumab) axes [62].

However, some epidemiological investigations introduce subtle caveats regarding cutaneous oncogenesis. In a large nationwide cohort study, researchers identified an association between IL-12/23 inhibitor use and an elevated risk for skin malignancies, with reported HR values ranging from 1.19 to 1.30 depending on the specific skin cancer outcomes evaluated, reflecting a degree of statistical uncertainty [47]. In contrast, large-scale registry data specifically tracking ustekinumab exposure displayed low incidence rates for BCC (0.1/100 PY) and cutaneous SCC (0.4/100 PY), coupled with a stable overall systemic malignancy rate of 2.6/100 PY [63]. In summary, while these data indicate that therapies targeting the IL-12/23 axes maintain a reassuring systemic safety profile, continued longitudinal screening remains urgent to clarify possible increases in skin cancer risks.

#### 5.3.3. Inhibitors of IL-17

IL-17 inhibitors directly target either the pro-inflammatory IL-17 cytokine ligand or their receptor subunits, serving as a highly effective therapeutic option for moderate-to-severe plaque psoriasis. Within this biological class, secukinumab and ixekizumab are fully human monoclonal antibodies that selectively bind and neutralize the IL-17A ligand. Concurrently, bimekizumab inhibits both IL-17A and IL-17F isoforms, while brodalumab is a human monoclonal antibody designed to target the IL-17Rα subunit, effectively blocking the signaling of multiple IL-17 isoforms [63,64]. This biological agent is mainly used in adult candidates for systemic or phototherapeutic interventions who have demonstrated primary or secondary therapeutic failure with alternative systemic regimens [64]. As a class, IL-17 inhibitors are characterized by an exceptionally rapid onset of clinical action, high rates of skin clearance (PASI_90_ and PASI_100_ responses), and a sustained therapeutic response over extended maintenance phases [58].

The long-term oncological impact of ixekizumab was evaluated in an integrated analysis encompassing 6892 patients with moderate-to-severe psoriasis followed over a 5-year period, with calculated IRs adjusted per 100 patient-years of exposure (PYE). Within this large-scale cohort, 58 individuals developed cutaneous malignancies, corresponding to a low overall skin cancer IR of 0.3/100 PYE. When stratified by specific histological types, the IR for NMSC was 0.3/100 PYE (occurring in 55 patients), which comprised a BCC IR of 0.2/100 PYE (44 patients) and a cutaneous SCC IR of 0.1/100 PYE (16 patients). Crucially, only two cases of melanoma were recorded (IR: 0.03/100 PYE). Clinical chart reviews indicated these cases were likely attributable to previous treatment-related confounding factors, such as extensive pre-baseline exposures to PUV_A_ or UV_B_ phototherapy, as well as conventional non-biological systemic therapies [65]. These findings support the long-term oncological safety of ixekizumab, indicating that extended therapeutic exposure does not drive an iatrogenic increase in treatment-related skin cancers [65].

Parallel oncological risk evaluations have been performed by the IL-17Rα antagonist brodalumab. An integrated analysis tracking psoriatic cohorts exposed to brodalumab for a minimum of 12 weeks demonstrated a low and reassuring long-term safety profile. The calculated incidence rate was 0.4/100 PY for BCC, 1.6/100 PY for cutaneous SCC, and 0.89/100 PY for non-NMSC malignancies, culminating in an overall malignancy rate of 0.9/100 PY. Additionally, very low organ-specific IRs were reported, including prostate adenocarcinoma (0.1/100 PY) and PC (<0.1/100 PY), while the low BCC IRs aligned closely with those documented for ixekizumab and secukinumab [63]. These findings are supported by a 5-year post-marketing pharmacovigilance data from the United States, which confirmed a stable, non-elevated non-NMSC malignancy rate of 0.89/100 PY in patients undergoing long-term brodalumab therapy [64]. An overview of psoriasis therapies and their reported associations with cancer risk are summarized in Table 2.

Treatment-associated malignancy risk must be interpreted within the broader clinical context of psoriasis rather than isolated to individual therapeutic agents. Decoupling the oncogenic effects of systemic inflammation from prolonged immunomodulation remains challenging, as patients on systemic therapies often exhibit more severe, long-standing disease and higher cumulative inflammatory burdens. While registries provide long-term safety data, they often lack complete records on cumulative drug exposure, history, treatment switching, and lifestyle confounders. Observational comparisons across therapeutic classes also suffer from indication bias. Though data suggest a more favorable oncological safety for IL-17 and IL-23 inhibitors over TNF-is, direct evidence is limited. Plus, because newer biologics are relatively recent, safety data extending beyond ten years remain unavailable. Therefore, clarifying all the aforementioned complex relationships will require future multicenter studies on cumulative treatment exposure and oncological outcomes.

## 6. Epidemiological Results for Psoriatic Patients Under Treatment: Links to Inflammatory Markers and Cardiovascular Disease

The clinical evaluation of contemporary therapeutic strategies in psoriasis extends far beyond assessing long-term oncological safety. An important aspect of patient management involves mitigating chronic systemic inflammation, a pathogenic driver that directly influences both cutaneous disease progression and CVDs. Recent studies have increasingly focused on the effects of anti-psoriatic therapies on circulating inflammatory markers, including hsCRP, NLR, PLR, and SII. These accessible markers are currently recognized as useful indicators of inflammatory activity, therapeutic response, and CVD risk in both clinical trials and routine dermatological practice.

### 6.1. Conventional Systemic Therapies and Inflammatory Markers

#### 6.1.1. Methotrexate (MTX)

Methotrexate (MTX) achieves its primary therapeutic effects by suppressing immune and inflammatory networks. However, baseline inflammatory statues can conversely influence long-term treatment persistence. Sugimoto et al. [44] evaluated the association between blood-derived inflammatory indices (NLR, MLR, PLR, SII, and SIRI), psoriasis severity, and drug survivability. Patients presenting with elevated platelet counts, PLR, and SII at treatment initiation exhibited notably lower MTX continuation rates. These findings indicate that increased inflammatory burden may negatively affect the efficacy of MTX and may be linked to treatment discontinuation or switching [44].

#### 6.1.2. Cyclosporine (CsA)

In the same study, the clinical baseline parameters of patients receiving CsA were systematically cross-examined. Individuals presenting with elevated baseline platelet and neutrophil counts, PLR, and SII experienced significantly lower drug survivability during treatment. Similar to the trends observed with MTX, these elevated cellular hematological indices appear to be strongly associated with reduced therapeutic efficacy in conventional non-biological options, which translates into a higher likelihood of premature drug discontinuation, secondary failure, or treatment modification [44].

#### 6.1.3. Acitretin (Act)

Albayrak [66] investigated changes in inflammatory biomarkers such as NLR, NMR, PLR, and SII levels in patients with plaque psoriasis. After three months of treatment, acitretin significantly reduced NMR and PLR following an initial 3 months of continuous treatment. However, unlike targeted systemic or biological modalities, acitretin’s improvements were not consistently maintained after six months, suggesting a more limited and less sustained anti-inflammatory effect [66].

### 6.2. Targeted Synthetic and Adjunctive Therapies and Inflammatory Markers

Targeted synthetic and adjunctive therapies have expanded the therapeutic landscape of psoriasis, especially for patients who respond inadequately to conventional systemic or biologic therapies. Beyond improving clinical symptoms, these approaches may also affect circulating inflammatory biomarkers, which are increasingly considered useful indicators of both disease activity and CVD risk.

#### 6.2.1. Inhibitors of Janus Kinase Inhibitors (JAKis)

Nilgün Şentürk [67] evaluated the therapeutic effects of JAKis and specifically tofacitinib, in patients with psoriasis. The study assessed both 5 mg and 10 mg doses and revealed that the 10 mg regimen achieved greater PASI_75_ than 5 mg responses in 40–64% of patients with moderate-to-severe psoriasis at 12 or 16 weeks. In addition, treatment safety was rigorously monitored up to week 28, while the vast majority of patients displayed sustained, optimal clinical improvement by week 52 [67].

Similarly, a systematic review and meta-analysis evaluating JAKis efficacy in moderate-to-severe psoriasis compared with placebo concluded that for tofacitinib, the overall PASI_75_ response reached an OR of 14.35 (95%, CI: 7.65–26.90), whereas for other JAKis the OR value was 6.42 (95%, CI: 4.89–8.43). When pooling data for all JAKis into an aggregate class-wide model, the overall PASI_75_ response yielded a highly robust OR of 9.88 (95%, CI: 8.13–12.00), confirming that this treatment class represents an exceptional therapeutic avenue for severe psoriasis [43]. Comparable findings were also reported for tofacitinib (TOFA) and baricitinib (BARI), whereas peficitinib (PEF) produced more modest clinical responses. Overall, JAKis provide good efficacy compared with placebo (mirroring or exceeding safety-to-efficacy trends established by TNF-α inhibitors in both RA and psoriasis models), while maintaining an acceptable, highly predictable safety profile across a broad spectrum of immune-mediated inflammatory diseases (IMIDs) [68].

#### 6.2.2. Dimethyl Fumarate (DMF)

Dimethyl fumarate (DMF) exerts antioxidant and anti-inflammatory effects through modulating multiple cellular pathways. In a recent clinical evaluation, DMF was deployed as adjunctive therapy, alongside IL-12/23 inhibitors in a small proportion of patients (2.33%). Combination treatment was associated with reductions in PASI and NLR scores, whereas alterations in PLR did not reach statistical significance. Although these findings suggest a potential anti-inflammatory benefit, the small number of treated patients limits firm conclusions, and larger prospective studies are required to clarify the relationship between DMF therapy and circulating inflammatory biomarkers [69].

### 6.3. Biological Treatments and Inflammatory Markers

#### 6.3.1. TNF-α Inhibitors

TNF-α inhibitors consistently reduce systemic inflammation in patients with psoriasis, as reflected by changes in several hematological inflammatory bio markers. Albayrak [66] reported decreases in NMR and PLR after three months of treatment with adalimumab, etanercept, and infliximab, while NLR declined significantly in patients receiving adalimumab and infliximab. Moreover, SII decreased only in the adalimumab treatment group. These improvements were consolidated after six months, with adalimumab showing the most consistent reductions across NLR, NMR, and SII, indicating sustained suppression of systemic inflammation [66].

Beyond monitoring treatment response, several studies have evaluated whether baseline inflammatory biomarkers predict biological drug efficacy. Sugimoto et al. [44] demonstrated that TNF-α inhibitor adalimumab achieved high drug survivability regardless of baseline NLR, PLR, and SII values, while treatment itself significantly reduced NLR and PLR over time [44]. Andersen et al. [46] further reported that following treatments with adalimumab, infliximab, certolizumab, and etanercept, responders exhibited significantly lower baseline NLR levels than non-responders [46]. Baseline IL-6 levels were also lower in responders, a trend that reached statistical significance with the adalimumab subgroup (IL-6: 0.99 pg/mL in responders vs. 1.62 pg/mL in non-responders, *p* = 0.02). Conversely, baseline PLR and SII values did not differ significantly between response groups, while circulating soluble IL-6 receptors (sIL-6Rs) tended to be higher in non-responders than responders (sIL-6R: 39.6 vs. 30.1 ng/mL, *p* = 0.06). These findings suggest that baseline NLR and circulating IL-6 levels may serve as accessible predictive biomarkers to stratify a patient’s likelihood of responding to TNF-α inhibition [46].

The anti-inflammatory effects of TNF-α inhibitors are further supported by longitudinal clinical studies. Isa An et al. [70] reported significant reductions in NLR, PLR, MPV, and CRP following biological therapy [70]. Similarly, Najar Nobari et al. [71] documented marked declines in NLR (from 4.76 ± 2.12 at baseline to 1.71 ± 0.93) and PLR (from 8.34 ± 3.40 to 4.28 ± 2.80) after 12 months of treatment with infliximab, adalimumab, or etanercept, accompanied by substantial improvements in PASI scores. Interestingly, elevated body mass index (BMI), smoking, and alcohol consumption were associated with persistently higher inflammatory indices, highlighting the contribution of modifiable CVD risk factors to systemic factors, indicating that lifestyle parameters compound the baseline inflammatory burden inflammation in psoriasis [71].

Similarly, Kommoss et al. [69] tracking IL-17, IL-12/23, IL-23, and TNF-α inhibitors including adalimumab and DMF, demonstrated that reductions in NLR closely paralleled improvements in PASI during biological therapy from 16.35 to 4.2 at week 4 and further down to 1.6 by week 16. PLR did not show significant variation. These findings reinforce NLR as one of the most responsive biomarkers for monitoring treatment efficacy and suggest that reductions in systemic inflammation may also translate into lower CVD risk [69]. Comparative registry data further indicate differences in clinical efficacy within the TNF-α inhibitor class. R.B. Warren et al. [72] reported PASI_90_ responses in 50.1% of adalimumab-treated patients compared with 20.1% of those treated with etanercept after six months, although male cohorts demonstrated slightly lower response rates than females [72].

Collectively, the available evidence demonstrates that TNF-α inhibitors reduce systemic inflammatory activity, particularly NLR and PLR, while baseline NLR and IL-6 offer clear prognostic value for predicting clinical response. Although individual biomarkers differ in their predictive performance, NLR emerges as the most reproducible indicator across studies. Because elevated NLR has also been associated with increased CVD risk, these findings suggest that successful TNF-α inhibition may provide benefits extending beyond skin clearance by minimizing the systemic inflammatory burden.

#### 6.3.2. IL-12/23 Inhibitors/IL-23 Inhibitors

##### IL-23 Inhibitors

Selective IL-23 inhibitors consistently reduce systemic inflammatory activity while providing sustained clinical improvement in patients with moderate-to-severe psoriasis. Recent studies have evaluated whether these therapeutic benefits are paralleled by changes in circulating inflammatory biomarkers associated with CVD risk. Recently, Andersen et al. [46] evaluated patients receiving ustekinumab, guselkumab, and risankizumab and found no significant differences in baseline NLR, PLR, or SII between responders and non-responders. However, the statistical power of this subgroup analysis was limited by the relatively small sample size [46].

However, longitudinal data from Sugimoto et al. [44] demonstrated that selective IL-23p19 inhibitors (guselkumab, tildrakizumab, and risankizumab) achieve notably high treatment continuation rates, maintaining drug survivability independently of a patient’s baseline inflammatory profile. Furthermore, continuous therapy with IL-23 inhibitors was associated with significant reductions in peripheral NLR and PLR over time, supporting the systemic anti-inflammatory effects of this therapeutic class [44].

These findings align with data from Kommoss et al. [69], where the aggregate systemic cohort achieved a median PASI reduction from 16.35 to 1.6 after 16 weeks of systemic therapy. Within the selective IL-23 inhibitor subgroup, this clinical clearance was accompanied by a robust, parallel decrease in NLR values, whereas PLR showed only a slight reduction and was not correlated with clinical response [69]. Additionally, Eyerich et al. [73] demonstrated that extending guselkumab maintenance dosing from 8 to 16 weeks in super-responders was non-inferior for maintaining disease control, further supporting the durability of IL-23 inhibition [73]. Overall, current evidence indicates that IL-23 inhibitors achieve durable clinical responses while simultaneously reducing systemic inflammatory activity, particularly NLR. These findings support the utility of NLR as a practical biomarker for monitoring therapeutic response and for potential CVD risk reduction during IL-23 inhibition.

##### IL-12/23 Inhibitor (Ustekinumab)

Ustekinumab, a monoclonal antibody targeting the shared *p40* subunit of IL-12 and IL-23 cytokines, has also been associated with reductions in circulating inflammatory biomarkers. In Albayrak’s study [66], ustekinumab reduced NMR and PLR after six months [66]. Similarly, the long-term biomarker evaluation by Isa An et al. [70] confirmed that all assessed biomarkers, including NLR, PLR, MPV, and CRP, followed a uniform downward trend over a 6-month follow-up period, suggesting that ustekinumab may contribute to the suppression of systemic tissue inflammation effectively [70].

More specifically, Taşkın et al. [74] examined the utility of serial laboratory monitoring in psoriatic patients managed with ustekinumab every 12 weeks. Their data showed significant reductions in NLR, dropping from 2.34 to 1.93 once patients achieved a PASI_75_ response. This was mirrored by a significant reduction in PLR, which dropped from 125.8 to a post-treatment median of 107.5. In contrast, absolute MPV and CRP did not alter significantly, suggesting that NLR and PLR represent more sensitive tools for assessing real-time disease severity and treatment response during ustekinumab therapy [74].

These findings are supported by Kommoss et al. [69], who noted a consistent downward tendency for NLR in matching the clinical PASI improvements in ustekinumab-treated patients, while PLR values remained statistically unchanged [69]. From a long-term clinical perspective, Warren et al. [72] reported that ustekinumab achieved a PASI_90_ response in 49.9% of patients at the 6-month mark and successfully maintained these clearance rates at 12 months, confirming its role as a stable, highly dependable long term treatment option [72]. Taken together, these findings indicate that ustekinumab provides sustained clinical efficacy while reducing systemic inflammatory activity, with NLR emerging as the most consistent laboratory marker of treatment response.

##### IL-17 Inhibitors

IL-17 inhibitors are among the most effective biologic therapies for psoriasis and consistently reduce systemic inflammatory activity alongside clinical disease severity. Several studies have evaluated their effects on circulating inflammatory biomarkers and their potential prognostic value. Sugimoto et al. [44], by evaluating the relationship between inflammatory markers, disease severity, and medication continuation rates, revealed that patients receiving IL-17 inhibitors exhibited high treatment continuation rates regardless of their baseline inflammatory profile. Treatment was associated with significant reductions in NLR and PLR while maintaining high drug survivability irrespective of baseline inflammatory status [44]. Andersen et al. [46] noted that for IL-17/IL-17Rα inhibitors, including secukinumab, ixekizumab, and brodalumab, no significant differences were observed in NLR, PLR, or SII between responders and non-responders. However, sIL-6ST levels were higher in responders than non-responders (sIL-6ST: 128 vs. 105 ng/mL, *p* = 0.02), suggesting a possible predictive role for this marker in treatment response [46].

In a dedicated evaluation of secukinumab in psoriasis vulgaris, Isa An et al. [75] documented significant reductions in absolute neutrophil and leukocyte counts. However, there was no statistically significant difference between NLR, PLR, and MPV before and after 4 months of treatment, suggesting that these specific metrics may have a more limited capacity to capture rapid inflammatory changes induced by secukinumab during short-term monitoring [75]. In contrast, longer-term data from Kommoss et al. [69] confirmed the value of NLR over an extended 16-week follow-up period. Within this study, where IL-17α inhibitors represented the most frequently prescribed biological intervention, the cohort’s median PASI decreased (16.35 to 1.6, 16 weeks). After 16 weeks, clinical improvement was accompanied by significant reductions in NLR, which positively correlated with disease severity, whereas PLR remained unchanged, indicating its limited prognostic value [69].

Current evidence identifies NLR as the most consistent inflammatory biomarker during IL-17 inhibition, whereas PLR appears less sensitive for monitoring therapeutic response. The reduction in NLR may reflect attenuation of the systemic inflammatory burden linked to increased CVD risk in psoriasis. An overview of the effects of psoriasis therapies on inflammatory biomarkers, clinical outcomes, and CVDs are summarized in Table 3.

### 6.4. Psoriasis Treatment and Cardiovascular Risk

The consistent reduction in inflammatory biomarkers observed across several therapeutic classes is clinically relevant because elevated NLR, PLR, SII, and hsCRP have all been associated with endothelial dysfunction, atherosclerosis, and future CVD events. Consequently, therapies that effectively suppress these biomarkers may provide CVD benefits extending beyond skin disease control.

Beyond achieving cutaneous clearance, contemporary psoriasis therapies aim to reduce the systemic inflammatory burden that contributes to CVD morbidity. Chronic systemic inflammation is now recognized as an independent driver of endothelial dysfunction, accelerated atherogenesis, myocardial infarction, stroke, and arterial (ATE) and venous thromboembolic events (VTEs) in psoriatic patients. Consequently, increasing attention has focused on whether anti-psoriatic therapies modify CVD risk through direct vascular effects or indirectly suppressing systemic inflammation. This section has summarized current epidemiological evidence regarding CVD safety and potential cardioprotective effects of major therapeutic classes [76]. The following section summarizes the epidemiological evidence linking anti-psoriatic treatments with CVD outcomes.

#### 6.4.1. JAK Inhibitors

JAKis have been scrutinized regarding their thromboembolic and CVD safety profiles across several immune-mediated inflammatory diseases (IMIDs). In a comprehensive epidemiological study by Setyawan et al. [76], individuals diagnosed with IMDs exhibited a notably higher baseline risk for thromboembolic complications compared to matched healthy controls, reporting an adjusted IRR of 1.49 (95%, CI: 1.40–1.59, *p* < 0.0001), This systemic elevation spanned multiple distinct vascular events, including deep vein thrombosis (DVT, IRR = 1.78), pulmonary embolism (PE, IRR = 1.66), acute myocardial infarction (MI, IRR = 1.17), and ischemic stroke (IS, IRR = 1.35, *p* < 0.05). When isolating the specific impact of JAKis within this high-risk population, a statistically significant increase in PE was identified (IRR = 2.52, 95%, CI: 1.11–5.70), alongside numerical but non-significant elevations in rates of DVT (IRR = 1.23) and IS (IRR = 1.82) [76].

To further contextualize these risks, Mease et al. [77] evaluated the long-term safety profile of tofacitinib administered at doses of 5 mg or 10 mg twice daily relative to active-comparator TNF inhibitors in a cohort with pre-existing CVD risk factors. Absolute IRRs for DVT, PE, total VTE, and ATE remained low overall. Explicitly, the calculated IRR for DVT was identical at 0.06 events per 100 PY for both the 5 and 10 mg cohorts. The IRR for PE was 0.13/100 PY in the 5 mg group and 0.09/100 PY in the 10 mg group. Total VTE rates tracked at 0.19/100 PY for the 5 mg and 0.15/100 PY for the 10 mg dose, respectively, demonstrating a minor reduction in relative VTE risk at the higher dose. In contrast, a paradoxical trend emerged for ATEs, where the IRR was higher in the lower-dose cohort, tracking at 0.52/100 PY with the 5 mg dose compared to 0.22/100 PY with the 10 mg dose [77]. Such findings indicate that while JAKis are highly effective therapeutic options, their CVD and thromboembolic safety profiles require proactive, individualized clinical monitoring. Although absolute event rates remained low, PE occurred more frequently among patients receiving JAKis, particularly those with pre-existing CVD risk factors.

#### 6.4.2. Systemic Glucocorticoids

The use of systemic glucocorticoids in psoriasis is generally discouraged due to the risk of triggering erythrodermic or pustular flares upon withdrawal. However, they are occasionally deployed for severe, co-morbid inflammatory states. In the large-scale multi-IMD meta-analysis by Setyawan et al. [76], the systemic administration of glucocorticoids was associated with markedly increased rates of DVT (IRR = 1.20, 95%, CI: 1.05–1.36), PE (IRR = 1.19, 95%, CI: 1.00–1.41), and MI (IRR = 1.21, 95%, CI: 1.00–1.46). Conversely, no statistically significant increase was observed for ischemic stroke [76]. These findings suggest that systemic glucocorticoids appear to increase CVD and thromboembolic risk and should therefore be used cautiously in patients with existing CVDs.

#### 6.4.3. Hormone Replacement Therapy

Although not a psoriasis-specific therapy, hormone replacement therapy (HRT) was evaluated because many women with psoriasis receive concomitant hormonal treatment. In contrast to the risks associated with systemic corticosteroids, HRT revealed a protective trend against specific thromboembolic outcomes within the same multi-IMD cohort [76]. Psoriatic patients received various treatments, including HRT, oral contraceptives, glucocorticoids, JAKis, and sphingosine 1-phosphate (S1P) receptor modulators. While the baseline cohort with IMD conditions carried an elevated overall thromboembolic risk (adjusted IRR = 1.49; 95%, CI: 1.40–1.59, *p* < 0.0001), the subgroup of women receiving concurrent HRT exhibited significant reductions in the relative IRR of DVT (IRR = 0.64), PE (IRR = 0.61) and IS (IRR = 0.56). Furthermore, no statistically significant correlation was identified between HRT and the incidence of MI within this population [76].

#### 6.4.4. S1P Modulators

Sphingosine 1-phosphate (S1P) receptor modulators represent emerging immunomodulatory agents evaluated across immune-mediated inflammatory diseases and prevent the egress of lymphocytes from lymphoid tissues. In the comprehensive, multi-IMD dataset, S1P receptor modulation demonstrated a distinct protective trend across multiple vascular endpoints [76]. This included a numerical reduction in the incidence of DVT (IRR = 0.61), PE (IRR = 0.30), and acute MI (IRR = 0.54), although due to sample size distributions, statistical significance within this drug class was achieved exclusively for the prevention of IS by 67% (IRR = 0.33) [76].

#### 6.4.5. Oral Contraceptives

Conversely, the administration of oral contraceptives as concomitant medications in women with underlying IMIDs was associated with an increased risk of venous thromboembolic complications. The meta-analysis documented a statistically significant elevation in DVT rates, yielding an IRR of 1.40 (95%, CI). Additionally, a numerical but statistically non-significant increase was observed for IS (IRR = 1.54; *p* = not significant (NS)). In contrast, oral contraceptive use was related with non-significant numerical decreases in the incidence of acute MI (IRR = 0.47, *p* = NS) and PE (IRR = 0.83, *p* = NS) [76].

#### 6.4.6. Statins

Beyond their primary lipid-lowering effects, statins (HMG-CoA reductase inhibitors) exert important pleiotropic, cardioprotective, and anti-inflammatory effects in patients presenting with psoriasis. Because dyslipidemia is highly prevalent among patients with psoriasis, statins may provide dual benefits by improving lipid profiles while attenuating inflammation-associated CVD risk. Garshick et al. [34] investigated the metabolic and biochemical effects of atorvastatin therapy by tracking plasma fatty acid profiles in a psoriatic cohort. The administration of atorvastatin achieved a 44% reduction in circulating low-density lipoprotein (LDL-C). Concurrently, it notably altered systemic fatty acid metabolism, driving a 14% increase in arachidonic acid (AA) levels and a 12% reduction in baseline linoleic acid (LA) levels. This metabolic shift was accompanied by the upregulated expression of fatty acid desaturase 1 and 2 (FADS1 and FADS2) enzymes, which are critical components of the AA synthesis cascade [34]. The results indicate that statins operate through mechanisms beyond routine lipid reduction in psoriasis, acting as direct modulators of inflammatory lipid metabolism and highlighting their pleiotropic cardioprotective role in reducing CVD risk in psoriatic patients.

#### 6.4.7. Systemic and Biologic Therapies

Advanced systemic and biological therapies also influence inexpensive hematological biomarkers that reflect systemic vascular status and predict long-term CVD events. Conic et al. [33] evaluated RDW and MPV as cost-effective, readily available screening markers to predict a psoriatic patients’ risks of acute MI, atrial fibrillation (AF), and chronic heart failure (CHF) [33]. In a small subgroup of patients receiving systemic or biological agents, successful clinical improvement in skin severity was accompanied by the normalization of elevated RDW values among responders. Conversely, RDW values remained increased in the non-responder group [33]. Therefore, these dynamics identify RDW as a responsive biomarker in psoriatic cohorts, revealing how localized cutaneous tissue inflammation can trigger an inflammatory response in a distant organ system like the bone marrow, which subsequently normalizes upon achieving clinical remission.

#### 6.4.8. Aspirin

Given that platelet hyper-activity accelerates atherothrombosis, Garshick et al. [35] characterized the baseline platelet phenotype in patients with psoriasis, measuring its contribution to vascular inflammation and evaluating the therapeutic utility of low-dose acetylsalicylic acid (aspirin). Patients with psoriasis displayed notably increased platelet activation characterized by enhanced platelet endothelial adhesion and overexpression of IL-8, IL-1β, and COX-2. Two weeks of low-dose aspirin (81 mg/day) markedly attenuated platelet-mediated endothelial inflammation. This intervention achieved a greater than 70% improvement in a composite endothelial inflammation index, alongside a robust reduction in circulating thromboxane B_2_ (TxB_2_) levels (7.8 ng/mL down to 1.0 ng/mL) [35]. These findings indicate that low-dose aspirin can effectively suppress platelet-mediated vascular inflammation in patients presenting with psoriasis.

#### 6.4.9. TNF-α Inhibitors

Finally, TNF-α inhibitors have been evaluated via large-scale pharmacovigilance frameworks to identify potential associations with adverse CVD events. Ma et al. [78] executed a disproportionality analysis tracking adalimumab, etanercept, infliximab, golimumab, and certolizumab pegol within global safety databases. Among these five agents, adalimumab alone generated positive signals for acute MI (reporting odds ratio (ROR) of 1.58 (95%, CI: 1.51–1.64) and general arterial thrombosis (ROR = 1.54, 95%, CI: 1.49–1.58). These findings were confirmed by an independent information component methodology, which yielded positive safety signals for general thrombosis (IC_025_ = 0.15), pulmonary thrombosis (IC_025_ = 1.72), MI (IC_025_ = 0.37), coronary artery occlusion (IC_025_ = 2.99), and cerebral thrombosis (IC_025_ = 2.99) within the finalized dataset [78].

In contrast, etanercept, infliximab, golimumab, and certolizumab pegol revealed negative risk signals across all evaluated standardized MedDRA queries (SMQs). Several agents demonstrated a protective profile, highlighted by certolizumab pegol, which generated a strong inverse signal for acute heart failure (IC_025_ = −6.88). Importantly, these pharmacovigilance findings were not consistent across the TNF-α inhibitor class. While adalimumab generated positive CVD safety signals, the remaining four TNF-α inhibitors generally showed neutral or even protective CVD profiles [78].

Psoriasis therapies exert heterogeneous effects on CVD or thromboembolic risk. Conventional systemic corticosteroids and certain JAKis may increase thromboembolic risk in susceptible individuals, whereas biologic therapies, statins, and low-dose aspirin reduce systemic inflammation and improve vascular function. Because CVD risk in psoriasis is multifactorial, treatment selection should incorporate both dermatological efficacy and the patient’s underlying CVD profile to maximize long-term clinical outcomes. An overview regarding the cardiovascular implications of several psoriasis therapies is summarized in Table 4.

Overall, the available epidemiological evidence suggests that psoriasis is associated with a higher risk of selected malignancies. However, the strength of these associations varies considerably based on cancer type and study design. MR studies provide stronger support for potential causal relationships by minimizing confounding variables, whereas observational studies primarily display statistical associations. Similarly, meta-analyses increase statistical power but frequently provide inconsistent findings and other factors including disease severity, lifestyle habits, and therapeutic exposure further complicate data interpretation. Overall, the current evidence supports a cautious interpretation in which both psoriasis-associated inflammation and external factors may contribute to cancer risk.

## 7. Epidemiological Outcomes When Targeting Modifiable Pathways in Psoriasis

Beyond pharmacological therapeutic approaches, increasing attention has been directed toward modifiable lifestyle factors such as diet, psychological stress, and metabolic health that may attenuate systemic inflammation and complement conventional psoriasis management. Dietary habits, body composition, metabolic health, and psychosocial well-being all influence inflammatory pathways and may contribute to disease activity, CVD risk, and reduced health-related quality of life (HR-QoL) [79]. Historically, the effect of dietary interventions has not been widely investigated in relation to the gut–skin axis, as the focus has mainly been confined to the skin itself. Nonetheless, various studies have recently been conducted to investigate the effects of different diets on psoriasis [80]. Although these interventions cannot replace standard pharmacological treatments, accumulating evidence suggests that they may provide meaningful adjunctive benefits by reducing systemic inflammation and improving patient-reported outcomes.

Thao Thi Nguyen et al. [79] evaluated the effects of a mind-body intervention (MBI) based on the relaxation response approach compared with treatment as usual (TAU) in psoriatic patients. During the 12-week intervention period and a subsequent follow-up at week 26, a significant difference in the WHO-5 Well-Being Index was observed between the two groups at week 12. Throughout the study period, the mean WHO-5 score was higher in the MBI group (65.3) than in the TAU group (59.1), suggesting that integrating a biopsychosocial intervention into the management of psoriatic patients may potentially improve health-related quality of life [79]. These findings suggest that MBIs may serve as valuable adjunctive therapies by improving psychological well-being and HR-QoL, although their direct effects on inflammatory biomarkers remain to be established.

Similarly, Silmara Meneguin et al. [81] explored patients’ perceptions of psoriasis in relation to their lifestyle to identify areas for improvement. Their findings showed that psoriasis affects quality of life not only because of disease severity, but also because of social stigma, altered self-image, and a pervasive feeling among patients that the actual burden of the disease is underestimated in daily clinical practice [81]. Psychological burden constitutes an important modifiable factor that should be addressed alongside pharmacological treatment to optimize long-term disease management.

Metabolic factors have also been extensively investigated in psoriatic disease. Tim Blake et al. [82], compared body composition parameters between patients with psoriatic disease and control groups. They found that several MRI-derived measures, such as total body volume, subcutaneous adipose tissue (SAT), visceral adipose tissue (VAT), abdominal adipose tissue (AAT), and calculated VAT/AAT, and VAT/SAT ratios, were notably higher in patients with psoriatic disease than in healthy controls [82]. These observations support the growing concept that psoriasis is closely associated with visceral adiposity and metabolic dysfunction, both of which contribute to chronic low-grade inflammation and increased CVD risk.

Dietary interventions have also attracted attention in the context of psoriasis, particularly regarding the potential implications of the gut–skin axis. Lynda Grine et al. [80], investigated the effects of a macrophage migration inhibitory factor (MIF) on skin, gut, and metabolic health in psoriatic patients. Following enrollment, participants were expected to record their dietary and exercise habits for two full weeks, and the intervention was planned to last for 12 weeks. Nevertheless, due to the disruptions caused by the COVID-19 pandemic outbreak, this study was prematurely discontinued and no final results were obtained [80]. Nevertheless, the study highlights the growing recognition of nutritional modulation as a promising research area, although robust clinical evidence supporting specific dietary interventions remains limited.

Overall, current evidence indicates that modifiable lifestyle factors influence psoriasis through multiple interconnected inflammatory and metabolic pathways. Psychological interventions consistently improve quality of life, while obesity and visceral adiposity are the main drivers of systemic inflammation and increased CVD risk. However, evidence supporting dietary interventions and direct comparisons between therapeutic strategies remains inconclusive due to differences in study design, patient characteristics, treatment duration, and biomarker assessment. Altogether, psoriasis management must combine pharmacological therapy with lifestyle modification to reduce systemic inflammation and improve long-term health outcomes. Large prospective studies are also required to establish the long-term clinical significance of these findings.

## 8. Comparisons of Psoriasis Treatment Options

Although individual therapeutic classes have demonstrated favorable effects on psoriasis severity, systemic inflammation, CVD outcomes, and malignancy risk, direct comparisons between treatment strategies remain limited. Comparative studies are nevertheless valuable because they help clinicians balance efficacy with long-term safety and identify therapies that provide the greatest overall benefit. This section summarizes the available evidence comparing biologic and non-biologic therapies with respect to inflammatory biomarkers, CVD outcomes, and cancer risk.

### 8.1. Comparison of Treatment Approaches Related to Inflammatory Markers

#### 8.1.1. Comparison Between Biological Therapies and Non-Biological Therapies

Comparative evaluation of therapeutic approaches in psoriasis highlights important differences in their effects on systemic inflammatory markers and treatment stability. A study by Silviu-Horia Morariu et al. [83] assessed the reliability of inflammatory markers derived from peripheral blood measurements, such as PLR, NLR, d-NLR, PMR, SII, and SIRI, in monitoring responses to different therapeutic categories. The treatments evaluated included TNF-α inhibitors (adalimumab, etanercept, infliximab, and certolizumab), IL-17 inhibitors (ixekizumab and secukinumab), IL-23 inhibitors (risankizumab, guselkumab, tildrakizumab, and ustekinumab), and non-biological/small-molecule inhibitors (apremilast), with measurements performed at 3, 6, and 12 months [83].

IL-17 inhibitors, demonstrated the strongest and most consistent reductions in inflammatory markers, particularly NLR and composite indices such as SIRI, and were associated with the highest response rates (PASI_90/100_). IL-23 inhibitors also showed significant reductions in inflammatory markers, although these effects appeared more gradual yet remained sustained over time. In contrast, TNF-α inhibitors showed moderate and less consistent effects across different inflammatory indices. Non-biological therapies, particularly apremilast, did not show significant changes in inflammatory markers, although the small sample size limits data interpretation [83].

Regarding treatment duration, a large reduction in inflammatory markers (PLR, d-NLR, SII, and SIRI) was observed in clinical responders (*p* < 0.05) after 6 months, whereas stabilization of systemic inflammation was noted after 12 months. Both IL-17 and IL-23 inhibitors drove significant changes in inflammatory markers such as NLR and SII, confirming their target effectiveness in reducing systemic inflammation. TNF-α inhibitors also demonstrated some efficacy, but their results were less consistent across the different drugs within the class. Finally, non-biological treatment with apremilast did not yield significant changes in any of the analyzed hematological markers [83]. A summary of the comparative effects of the different treatment categories on inflammatory markers and clinical response is presented in Table 5.

These findings are supported by Sugimoto et al. [44], who followed the same study pattern. Patients were categorized based on the initial treatment regimen they commenced, which included topical, phototherapy, non-biological (etretinate, apremilast, cyclosporine, and methotrexate) and biologic therapies, including TNF-α (infliximab and adalimumab), IL-17 (secukinumab, ixekizumab, and brodalumab) and IL-23p19 inhibitors (guselkumab, tildrakizumab, and risankizumab) [44].

Patients receiving conventional non-biological therapies exhibited lower continuation rates when baseline inflammatory markers, particularly PLR and SII, and absolute neutrophil count were elevated, suggesting reduced efficacy or tolerability in highly inflammatory states [44]. In contrast, patients receiving biological therapies demonstrated high treatment continuation rates regardless of their baseline inflammatory profile, accompanied by significant longitudinal reductions in NLR and PLR during the course of treatment [44]. Therefore, this study exhibited that the administration of biological therapy was more effective and provided greater therapeutic stability within a highly inflammatory environment. Although the study was limited by its small sample size and a one-year, single-center follow-up, the data suggest that a personalized approach is necessary when managing systemic inflammatory conditions [44].

#### 8.1.2. Comparison Between JAK and TNF-α Inhibitors

Comparative data between JAKis and TNF-α inhibitors remain limited. However, Nilgün Şentürk [67] evaluated the effects of tofacitinib in patients with psoriasis and reported similar clinical outcomes between tofacitinib (10 mg) and etanercept, a TNF-α inhibitor [67]. Although JAKis are not widely approved for psoriasis, these observations underscore the need for further research into their long-term clinical reliability.

### 8.2. Comparison Between Biological Therapies Related to Inflammatory Markers

Isil Deniz Oguz and Sevgi Kulakli [84] analyzed changes in inflammatory markers, particularly NLR, PLR, MPV, PCT, and CRP, associated with systemic inflammation and CVD risk, before and after biological treatment in patients with chronic plaque psoriasis. Patients were divided into four groups according to the biological therapy received, which included IL-12/23 (ustekinumab), TNF-α (adalimumab and certolizumab), IL-17 (ixekizumab and secukinumab), and IL-23 (guselkumab and risankizumab) inhibitors. Treatment effects were clinically evaluated three months after initiation [84].

Statistically significant reductions in absolute neutrophil counts, platelet counts, NLR, PLR, PCT, and CRP values were observed across the patient cohort. In contrast, no significant changes were observed in absolute lymphocyte counts or MVP values when comparing post-treatment levels to baseline. These findings indicate that biologic therapies, regardless of their specific molecular mechanism of action, exert a beneficial effect in mitigating systemic inflammation and reducing the risk of CVD morbidity in psoriatic patients. In conclusion, NLR and PLR are considered effective, sensitive parameters for monitoring systemic inflammation, whereas MPV was not a reliable indicator for determining the severity of inflammation in this setting [84].

Overall, the study supports the clinical utility of NLR and PLR as valuable markers for monitoring systemic inflammation during biologic treatment, whereas MPV did not appear to be a dependable indicator of inflammatory intensity (Table 6). However, the lack of differentiation between individual agents within each therapeutic category limits the assessment of their relative, head-to-head efficacy. In addition, the small sample size and short follow-up period must be taken into account when interpreting these findings [84].

### 8.3. Comparison of Non-Biological Therapies

#### Comparison Between JAK Inhibitors and PDE4 Inhibitors

Hidehisa Saeki et al. [85] evaluated the efficacy of deucravacitinib, a selective TYK2 inhibitor belonging to the JAKi class, in patients presenting with moderate-to-severe plaque psoriasis. This small-molecule agent was assessed across two identical, multinational Phase III studies (POETYK PSO-1 and POETYK PSO-2), where it was directly compared against the oral phosphodiesterase 4 (PDE4) inhibitor, apremilast [85].

The therapeutic outcomes demonstrated that in the POETYK PSO-1 trial, deucravacitinib achieved a 75% reduction in PASI_75_ in 58.4% of patients compared to 35.1% of those managed with apremilast. Concurrently, a static Physician’s Global Assessment score of clear or almost clear (sPGA 0/1) was achieved by 53.6% and 32.1% of patients, respectively. Consistent efficacy patterns were reported in the POETYK PSO-2 trial, where PASI_75_ reduction was achieved in 53.0% of patients receiving deucravacitinib, compared with 39.8% in the apremilast group and 9.4% in the placebo group. Regarding higher thresholds of skin clearance, PASI_90_ response rates tracked at 27.0% for deucravacitinib and 18.1% for apremilast, whereas sPGA 0/1 scores were achieved by 49.5% and 33.9% of the respective treatment arms. Collectively, these comparative data indicate that the TYK2 inhibitor deucravacitinib (JAKi) appeared to be more effective than the PDE4 inhibitor apremilast in inducing objective clinical responses across both PASI and sPGA endpoints [85].

Overall, these findings suggest that TYK2 inhibition provides superior clinical efficacy compared with PDE4 inhibition, supporting its emergence as a promising oral targeted therapy for moderate-to-severe psoriasis.

### 8.4. Comparison of Treatment Approaches Related to Cancer Risk

#### 8.4.1. Biological Versus Non-Biological Systemic Therapy

A recent cohort study by Tsai et al. [60] evaluated the association between the administration of biological versus non-biological systemic therapies and the subsequent risk of developing hematological malignancies in patients with psoriasis. Compared to a control cohort receiving conventional non-biological treatments (csDMARDs or nonsteroidal anti-inflammatory drugs (NSAIDs)), patients managed with advanced biological agents exhibited an elevated risk of lymphoid malignancies. Specifically, biological therapy was linked to an increased risk of lymphoma (adjusted IRR = 2.78, 95%, CI: 1.50–5.18, *p* < 0.01) and NHL (adjusted IRR = 2.48, 95%, CI: 1.28–4.80, *p* = 0.01). The baseline risk for lymphoid malignancies as an aggregate class was also significantly elevated (adjusted IRR = 1.78, 95%, CI: 1.08–2.93, *p* = 0.02) [60].

In contrast, no statistically significant increase was observed for overall hematological malignancies (adjusted IRR = 1.06, *p* = 0.91) or for myeloid neoplasms within the biological treatment arm. Notably, temporal stratification revealed that this excess risk related to biological interventions was most pronounced during the second year of active treatment (IRR = 2.95, 95%, CI: 1.14–7.67, *p* = 0.03), after which the relative incidence declined. These epidemiological findings underscore the clinical importance of implementing structured oncology surveillance during biological treatment, particularly during the second year of exposure, and support the need for further mechanistic research to clarify the pathways linking biological therapies with lymphomagenesis in psoriatic cohorts [60].

##### JAK Inhibitors Versus TNF-α Inhibitors

Russell et al. [55] investigated the long-term risk of malignancy associated with JAKis compared to both TNFis and MTX in psoriatic adults. Based on pairwise meta-analyses, treatment with JAKis was correlated with a notably higher overall cancer risk, including NMSC, when compared directly to TNFis (IRR = 1.50, 95%, CI: 1.16–1.94). The incidence of malignancies excluding NMSC was also higher with JAKis (IRR = 1.34, 95%, CI: 0.99–1.82), though this finding demonstrated borderline statistical significance [55].

With respect to organ-specific oncological effects, JAKis were associated with an increased risk of lymphoid malignancies, encompassing both Hodgkin and NHL, compared to TNFis (IRR = 1.48). When isolating individual agents within the class, tofacitinib was associated with a significantly higher incidence of NMSC than the active TNFis (IRR = 2.12, 95%, CI: 1.32–3.41), while an increased risk of LC was also recorded for general JAKi exposure relative to TNFis (RR = 1.82) [55]. Conversely, network meta-analyses comparing JAKis to conventional MTX therapy did not reveal a statistically significant variation in overall malignancy risk, including NMSC (IRR = 0.77, 95%, CI: 0.35–1.68). Interestingly, TNFis were found to be associated with a lower incidence of malignancy when compared to placebo (IRR = 0.47, 95%, CI: 0.28–0.81) [55]. Overall, the investigators concluded that while JAKis carried an increased risk of malignancy compared to TNFis, this elevated risk profile does not extend to comparisons with placebo or MTX. However, ongoing long-term registries remain essential to fully characterize the safety profile of these therapies over extended periods of use [55]. A comparison of the malignancy risk linked to JAKi versus TNFi use in psoriasis is summarized in Table 7.

##### Comparison Among Biological Therapies: TNF-A vs. IL-17 and IL-23 Inhibitors

A recent international population-based study investigated whether IL-17 and IL-23 inhibitors are associated with malignancy risk in patients with psoriasis receiving biologic therapies. More specifically, patients treated with IL-17 or IL-23 inhibitors were compared directly with those receiving TNF-α inhibitors. According to the findings, IL-17 inhibitors were associated with a lower risk of NHL (HR = 0.58), BCC (HR = 0.57), and melanoma (HR = 0.52) compared with TNF-α inhibitors. Similar patterns were observed for IL-23 inhibitors, which were associated with a reduced risk of NHL (HR = 0.39) and BCC (HR = 0.76) compared with TNF-α inhibitors. Overall, these results suggest that IL-17 and IL-23 inhibitors may be correlated with a more favorable malignancy risk profile than TNF-α inhibitors in patients with psoriasis. Compared with conventional systemic therapy, biologic agents were associated with a higher incidence of lymphoid malignancies, particularly lymphoma and NHL, whereas no increase was observed for overall hematological or myeloid malignancies. The comparative findings are summarized in Table 8 [86].

#### 8.4.2. Comparative Cardiovascular Evidence

##### Comparison Between Biological and Non-Biological Therapies

Philip Mease et al. [77] compared the TNF-α inhibitor adalimumab with the JAKi tofacitinib in relation to thromboembolic and CVD outcomes in patients with psoriasis. Overall, the IRs of DVT, PE, and VTE were generally similar between patients receiving tofacitinib and those treated with adalimumab, suggesting a broadly comparable safety profile for these specific outcomes [77].

A noticeable difference in results was observed regarding ATE, where the IR was significantly higher for patients receiving tofacitinib 5 mg (0.52/100 PY) compared to those receiving adalimumab, which demonstrated a lower value [77]. However, the 10 mg tofacitinib dose exhibited a lower IR (0.22/100 PY) compared to adalimumab. This renders them comparable in most cases, with the tofacitinib 10 mg having a slightly better safety profile in terms of arterial events compared to tofacitinib (5 mg) and adalimumab [77].

##### Comparison Between Non-Biological Therapies

In a 3-month study by Paulina Kiluk et al. [32], systemic treatments with acitretin (0.5 mg/kg/day) and MTX (15 mg/week) were compared in patients with psoriasis regarding their effects on endocrine growth factors, namely fibroblast growth factors 21 and 23 (FGF21 and FGF23), which may reflect metabolic and inflammatory status. Prior to treatment, FGF21 levels were notably elevated compared to healthy controls, especially in patients presenting with severe disease symptoms, dyslipidemia, or elevated CRP levels [32].

After 3 months of treatment, those receiving acitretin showed an almost threefold reduction in FGF21 levels, decreasing from a median of 37.7 to 12 pg/mL, (*p* < 0.05), whereas no drastic changes were observed in FGF23 levels. In addition, the baseline correlations between FGF21 and CRP, as well as the inverse correlation with cholesterol, were no longer evident after treatment. In contrast, patients treated with MTX did not show marked changes in either FGF21 or FGF23 after the same treatment period [32]. These results suggest that FGF21 might serve as a marker of metabolic inflammation in psoriasis. While acitretin appears to exert an additional metabolic effect beyond skin improvement, MTX remains an effective treatment, possibly operating through different inflammatory pathways. As for FGF23, its levels were not affected by either treatment option [32].

## 9. Other Studies Supporting the Psoriasis-Induced Inflammatory Link to Carcinogenesis

Psoriasis is a chronic, immune-mediated inflammatory disease characterized by persistent activation of innate and adaptive immune responses [87]. Dysregulation of the TNF-α, IL-23, and IL-17 cytokine axis sustains chronic inflammation through continuous activation of DCs, Th17 cells, and keratinocytes, generating a self-perpetuating inflammatory loop [88,89]. Increasing evidence indicates that this inflammatory state extends beyond the skin [87], contributing to systemic immune dysfunction and multiple comorbidities, including CVD disease and cancer [88,89]. In this context, the IL-23/IL-17 axis is a central molecular pathway linking chronic inflammation to the sustained activation of the immune system and subsequent disease progression [90].

### 9.1. Shared Cytokine Networks and Immune-Signaling Pathways Between Psoriasis and Cancer

The link between psoriasis and cancer was first proposed in the 1970s [91]. As mentioned earlier, this inflammatory disease is primarily caused by Th1, Th17, Th22, and Treg lymphocytes, along with cytokines such as IL-17, IL-6, (IL)-12, IL-22, IL-23, TNF-α, and various chemokines, and is characterized by an altered Th17/Treg balance [91,92]. Recent findings indicate that psoriasis-associated immune dysregulation extends beyond a simple Th17/Treg imbalance. Since inflammatory tissue-resident T-cell populations can persist even in clinically resolved lesions, thereby maintaining a local inflammatory memory that could contribute to disease recurrence [93].

In psoriatic skin, stressed keratinocytes contribute to the initiation and amplification of local inflammation by releasing pro-inflammatory cytokines, including TNF-α, IL-1, and IL-6, which promote DC cell activation. Activated DCs subsequently drive the differentiation of naïve T cells toward the Th1 and Th17 phenotypes through IL-12 and IL-23, respectively. These T-cell subsets then produce additional pro-inflammatory mediators, including TNF-α, IFN-γ, and IL-17, thus sustaining a self-amplifying inflammatory loop that promotes keratinocyte hyperproliferation and epidermal dysregulation [94].

TNF-α is considered a highly complex cytokine that is critical to the pathogenesis of psoriasis [95], a fact directly reflected by the corresponding therapeutic strategies targeting this specific signaling molecule. It is produced by a wide variety of cells, including keratinocytes, DCs, neutrophils, and mast cells, as well as natural killer T cells (NKT), Th1, Th17, and Th22 cells [87]. TNF-α amplifies inflammatory signaling by inducing the expression of additional downstream cytokines and chemokines through binding to TNFR1 and TNFR2, activating nuclear factor-κB (NF-κB) and mitogen-activated protein kinase (MAPK) signaling cascades that regulate inflammatory responses, cell survival, proliferation, and, under certain conditions, programmed cell death (apoptosis) [94].

The cytokines IL-23 and IL-17 have also been confirmed to markedly affect chronic inflammation. In general, IL-23 is involved in the activation of Th17 cells to induce the production of IL-17A, IL-17F, TNF, and IL-6 [96]. IL-17 induces the downstream gene expression by stimulating the activation of multiple signaling pathways, including canonical NF-κB, the CCAAT/enhancer-binding protein (C/EBP) family, and the MAPK pathway. Persistent IL-17 signaling activates NF-κB and MAPK pathways, promotes keratinocyte proliferation, and sustains chronic inflammation [96]. Because IL-17A also enhances anoikis resistance in triple-negative breast cancer (TNBC), this pathway may mechanistically link psoriasis-associated inflammation with tumor survival [95].

In addition to extracellular matrix (ECM) cytokine networks, intracellular signaling pathways, such as the JAK/STAT axis further reinforce the link between psoriasis, chronic inflammation, and cancer. JAK-STAT signaling regulates a variety of cellular functions, including proliferation, migration, differentiation, and apoptosis. Cytokines such as interferons, interleukins, and growth factors, along with their respective receptors, serve as the primary activators of the JAK pathway. In particular, interferons are known to influence psoriasis through the direct activation of the JAK/STAT signaling cascade [95].

Upon ligand binding, the receptor-ligand complex activates receptor-bound JAKs, which catalyze the phosphorylation of the receptor tyrosine [54]. JAK1, JAK2, JAK3, and TYK2, which are members of the JAK family, interact with specific cytokine receptors to recruit distinct STAT proteins. These proteins subsequently translocate to the nucleus to regulate the transcription of genes involved in inflammation, proliferation, differentiation, and cell survival [54,97,98]. TYK2, in particular, plays a crucial role in immune and inflammatory responses to the IL-6, IL-10, IL-12, and IL-23 cytokine families, as well as type I/II IFNs. Its dysregulation or deficiency can contribute significantly to the development of inflammatory diseases and AIDs [56,57].

Persistent activation of STAT1, STAT2, and STAT3, along with their activated (phosphorylated) variants, has been reported in patients with active psoriatic skin lesions [97]. Elevated IL-6 levels have also been reported in chronic inflammatory conditions, as well as in a wide range of hematopoietic malignancies and solid tumors, highlighting the potential relevance of IL-6-driven JAK/STAT signaling in the mechanistic overlap between psoriasis and cancer. Moreover, the IL-6/JAK2/STAT3 signaling pathway is aberrantly activated in many malignancies and is considered to contribute not only to tumor-promoting inflammatory signaling but also to the active suppression of anti-tumor immune responses [98]. Therefore, dysregulated JAK/STAT signaling, particularly via the excessive activation of STAT3, enhances anoikis resistance in tumor cells, whereas its silencing reduces anoikis resistance in melanoma and PC models [95].

Taken together, cytokine-driven pathways, particularly NF-κB and STAT3 signaling, contribute not only to chronic systemic inflammation in psoriasis but also to the establishment of a pro-tumorigenic microenvironment by promoting cell survival, proliferation, angiogenesis, cytokine production, and metastasis. Persistent activation of these pathways may shift chronically inflamed tissues toward a precancerous state [91,99].

### 9.2. Oxidative Stress, Keratinocyte Dysregulation, and Cancer-Related Signaling

Psoriasis is characterized by persistent oxidative stress, which represents a key mechanistic link between chronic inflammation to carcinogenesis. Oxidative stress results from an imbalance between the production of reactive oxygen species (ROS) and antioxidant defense systems, leading to oxidative damage of lipids, proteins, and DNA [100]. Elevated ROS levels have been consistently detected in psoriatic skin, suggesting a prolonged redox imbalance within the affected tissues [100].

Beyond causing direct cellular damage, ROS act as critical secondary messengers that activate multiple pro-inflammatory and pro-survival signaling pathways. In psoriatic skin, oxidative stress stimulates NF-κB, MAPK, and JAK/STAT signaling, thereby sustaining chronic inflammation and uncontrolled cell proliferation [101,102]. NF-κB promotes the transcription of pro-inflammatory cytokines such as TNF-α, IL-6, and IL-8, whereas STAT3 signaling promotes the survival, proliferation, and resistance of keratinocytes to apoptosis, features observed in both psoriasis and cancer malignancies [103,104].

Keratinocyte dysfunction is a hallmark of psoriasis and constitutes a critical bridge to carcinogenesis. Keratinocytes in psoriatic skin exhibit hyperproliferation, impaired differentiation, and resistance to programmed cell death, closely resembling a pre-neoplastic cellular state [105,106]. Cytokines such as IL-17, IL-22, and IL-6 activate intracellular pathways (e.g., STAT3 and PI3K/AKT), increasing the expression of cyclins and anti-apoptotic proteins, thereby promoting uncontrolled progression of the cell cycle [104,106]. This prolonged proliferative signaling is amplified by the activation of the MAPK and NF-κB pathways, creating a self-perpetuating inflammatory loop [101,102].

Oxidative stress also contributes to genomic instability, a key factor in carcinogenesis. ROS induce DNA strand breaks and base modifications, and when coupled with impaired DNA repair mechanisms, increase the likelihood of oncogenic mutations accumulating within chronically inflamed tissues [100,102]. Finally, oxidative stress promotes epigenetic dysregulation by altering DNA methylation patterns through inhibition of DNA methyltransferases, leading to aberrant expression of genes involved in inflammation and proliferation. For example, hypomethylation of SOCS3 reduces negative regulation of the JAK/STAT pathway, further enhancing inflammatory and proliferative signaling [107].

### 9.3. Platelet Activation, Endothelial Dysfunction, and PAF-Related Inflammatory Signaling

The inflammation associated with psoriasis, beyond cytokine-mediated immune dysregulation, also extends to vascular and thrombo-inflammatory abnormalities. Studies have shown that psoriatic patients have significantly higher levels of oxidation-modified lipids (OMLs), including oxidized LDL (ox-LDL), HDL (ox-HDL), and lipoprotein(a), ox-Lp(a), which promote a proatherogenic lipoprotein profile and impair HDL-cholesterol efflux capacity (CEC). In particular, ox-LDL directly induces endothelial cell dysfunction and atherosclerotic plaque formation [108]. In parallel, platelets are increasingly recognized as active mediators of immune and inflammatory processes. Platelet dysfunction contributes to psoriasis pathogenesis through the release of IL-1β, a cytokine that promotes tissue inflammation [108]. Furthermore, platelet activation facilitates the formation of platelet–lymphocyte aggregates and modulates IL-17 production, further amplifying inflammatory signaling [109].

Within this context, platelet activating factor (PAF) is recognized as a potent lipid inflammatory mediator linked to platelet activation, endothelial dysfunction, and the persistent presence of a thrombo-inflammatory environment. PAF and PAF-like agonists signal through the PAF receptor (PAFR), which when upregulated, mediates endothelial activation, elevated vascular permeability, and the recruitment of leukocytes and platelets. These processes lead to persistent endothelial dysfunction, leukocyte recruitment, and chronic vascular inflammation [109].

Along these lines, PAF signaling contributes to a cascade that produces several cytokines, growth factors, prostaglandins, adhesion molecules, and proteases. This creates a positive feedback loop that allows inflammation to continuously persist. Many of these identical processes are implicated in tumor pathophysiology. Based on the current literature, signaling through the PAF/PAFR axis can induce endothelial cell proliferation and migration, ECM breakdown, neoangiogenesis, and complex endothelial–inflammatory–tumor cell crosstalk. Tumor cells can also produce PAF and express PAFR, creating autocrine and paracrine loops that potentiate tumor proliferation and metastatic behavior. Collectively, the PAF/PAFR axis represents a potential mechanistic link between inflammation, endothelial dysfunction, platelet activation, angiogenesis, and carcinogenesis [110,111].

### 9.4. Mechanistic Synthesis of the Psoriasis-Cancer Inflammatory Link

The inflammatory pathways activated in psoriasis operate as an interconnected inflammatory network rather than as isolated mechanisms. Persistent signaling through TNF-α, IL-17, IL-23, and IL-6 sustains chronic immune activation, while downstream pathways including NF-κB, MAPK, and JAK/STAT promote inflammatory gene expression, cell survival, proliferation, and resistance to apoptosis [91,92,95,99]. Oxidative stress further amplifies these responses by causing genomic instability and keratinocyte dysfunction [100,102], whereas platelet activation, endothelial dysfunction, and thrombo-inflammatory mediators lead to vascular remodeling and aberrant angiogenesis [110,111].

**Figure 4:**
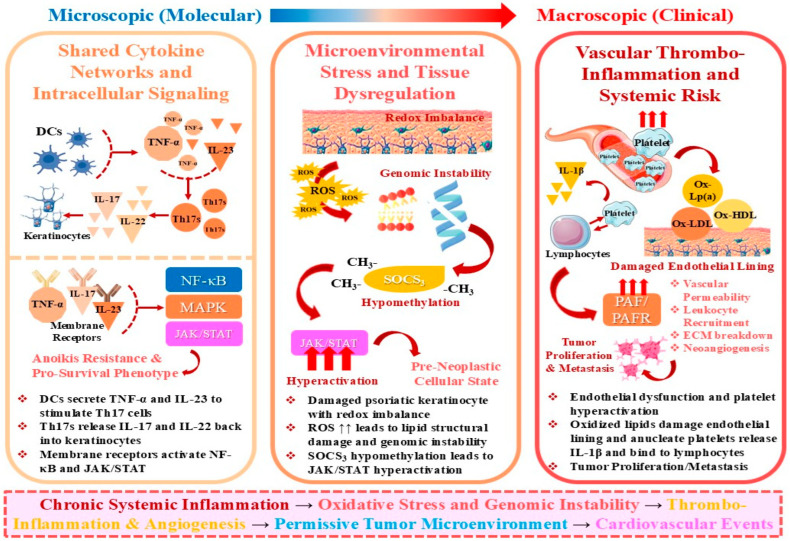
The psoriatic inflammatory continuum: molecular pathways, thrombo-inflammation, and the pathological link to carcinogenesis and cardiovascular risk. The schematic illustrates three integrated axes of progression: (**Left**) DCs secrete TNF-α and IL-23 to stimulate Th17 cells, creating an inflammatory loop with keratinocytes via IL-17 and IL-22, while triggering membrane receptor pathways (NF-κΒ, MAPK, and JAK/STAT) to promote anoikis resistance; (**Center**) ROS accumulation drives redox imbalance, lipid damage, and genomic instability, while SOCS3 hypomethylation leads to JAK/STAT hyperactivation and a pre-neoplastic cellular state; (**Right**) oxidized lipids (Ox-LDL, Ox-HDL, and Ox-Lp(a)) damage the endothelial lining, promoting platelet–lymphocyte interactions, IL-1β release, and PAF-PAFR activation. This drives vascular permeability, leukocyte recruitment, ECM breakdown, and neoangiogenesis, ultimately fostering a permissive microenvironment for tumor proliferation, metastasis, and adverse cardiovascular events.

Psoriasis involves a chronic inflammatory state capable of progressively remodeling the tissue microenvironment into one that favors tumor growth. Rather than arising from a single molecular pathway, the association between psoriasis and cancer reflects the cumulative effects of persistent inflammation, oxidative stress, immune dysregulation, endothelial dysfunction, and microenvironmental remodeling, which together may promote tumor initiation, progression, and metastasis. These molecular pathways, along with their links to thrombo-inflammation, carcinogenesis, and CVDs, are summarized in Figure 4.

## 10. In Vivo Studies Against the Psoriasis–Cancer Inflammatory Link

In addition to epidemiological and mechanistic evidence, several in vivo investigations in mouse models further elucidate the complex inflammatory pathways involved in psoriasis. The majority of these studies utilized topical imiquimod (IMQ) to induce a cutaneous phenotype that closely mirrors human psoriasis.

In one such study, Salamah et al. [112] administered IMQ to induce psoriasis-like lesions in order to evaluate the impact of psoriatic inflammation on platelet function and systemic hemostasis. The investigators reported significant platelet activation and enhanced systemic hemostatic activity, evidenced by increased fibrinogen binding and surface P-selectin expression, together with mCRAMP peptide levels in both plasma and skin. Furthermore, plasma isolated from psoriatic mice successfully activated platelets in healthy control mice, a phenomenon likely mediated via formyl peptide receptor 2 (FPR2)/lipoxin A4 receptor (ALX) receptor signaling. These findings strengthen the mechanistic link between psoriasis and a systemic prothrombotic state, indicating that it is driven, at least in part, by cathelicidin-mediated inflammatory pathways [112].

Further in vivo evidence underscores the importance of signaling pathways in driving hyperproliferation, angiogenesis, and inflammatory amplification. Ruijie Chen et al. [113] evaluated the therapeutic efficacy of chitosan/hyaluronic acid nanoparticles loaded with alantolactone (CHALT) in an IMQ-induced psoriasis mouse model. Topical application of CHALT led to a distinct clinical improvement in the psoriatic lesions, characterized by the inhibition of epidermal hyperproliferation, suppression of local angiogenesis, downregulation of STAT3 signaling, and a general attenuation of the local inflammatory response. These findings indicate that CHALT can deliver targeted, localized anti-inflammatory and antiproliferative activities [113].

The role of vascular regulation has also been rigorously examined using genetically modified mouse models. In an investigation exploring the function of the aryl hydrocarbon receptor (AhR) within vascular endothelial cells (VECs) in the skin, the selective depletion of endothelial AhR markedly exacerbated psoriasiform dermatitis induced by either IMQ or IL-23. The loss of AhR signaling in cutaneous VECs led to a pronounced worsening of psoriatic lesions, accompanied by dense neutrophil infiltration, microabscess formation within the stratum corneum, and marked vascular dilation. This pathological exacerbation was attributed to the increased expression of endothelial chemokines and adhesion molecules, resulting from the loss of normal AhR-mediated gene activation. Consequently, these data indicate that endothelial AhR exerts a protective effect by restricting inflammatory infiltration and microvascular dysregulation during psoriasis-like tissue inflammation [114].

Complementary murine studies have similarly shown that the pharmacological targeting of discrete inflammatory pathways can effectively mitigate psoriasis-like skin disease. Specifically, research by Jihye Kim et al. [115] revealed that administration of the *p300*/CBP inhibitor A485 effectively reduced tissue inflammation in mice with psoriasis-like skin lesions. This intervention led to reductions in both epidermal thickness and modified PASI-like scores. The sole documented adverse effect was transient weight loss, which completely resolved following the cessation of the treatment [115]. Likewise, Izumi Tsukayama et al. [116] demonstrated that malabaricone C suppressed psoriasis-like inflammation via the direct inhibition of the 5-lipoxygenase enzyme [116].

Another study evaluated the pathogenic role of the transient receptor potential ankyrin 1 (TRPA1), an ion channel involved in sensory and inflammatory cascades, in psoriasiform skin inflammation. In an IMQ-induced psoriasis model, TRPA1 knockout (KO) mice exhibited significantly diminished skin inflammation, reduced epidermal thickening, and impaired neutrophil recruitment, thereby supporting a pro-inflammatory role for TRPA1 in psoriasiform dermatitis [117].

Collectively, these murine models demonstrate that psoriasis-associated inflammation extends beyond keratinocyte hyperproliferation to encompass platelet activation, endothelial dysfunction, angiogenesis, neutrophil recruitment, and dysregulated cytokine signaling. As such, they provide valuable transitional frameworks for elucidating the mechanisms linking psoriasis with CVD and carcinogenesis, while supporting the development of targeted anti-inflammatory therapies.

## 11. Discussion: Challenges, Limitations, and Future Perspectives

This review synthesizes current epidemiological, mechanistic, translational, and therapeutic evidence supporting psoriasis as a systemic inflammatory disease associated with increased risks of selected malignancies and cardiovascular comorbidities. Psoriasis is a chronic, relapsing cutaneous AID characterized by profound systemic inflammation, immune-mediated pathology, and a complex multifactorial etiology. Driven by genetic susceptibility together with environmental and lifestyle stressors, its global incidence continues to rise [7,11]. Beyond its cutaneous manifestations, psoriasis severely compromises patients’ physical and psychosocial well-being due to highly prevalent comorbidities, such as major depressive disorder, CVDs, and psoriatic arthritis [49].

Epidemiological interest regarding a definitive link between psoriasis and malignancy has grown significantly. Documented associations encompass malignancies of the UDS tract, LIC, LC, pancreas, and urinary tract, alongside NHL, SCC, and BCC [49]. However, distinguishing the intrinsic oncogenic effects of chronic inflammation from the carcinogenic potential of conventional systemic therapies such as CsA, MTX, and PUV_A_ phototherapy remains challenging [63].

### 11.1. Epidemiological Landscape and Population Stratification

This comprehensive review is organized into two primary analytical domains: first, evaluating the direct association between psoriasis-induced autoimmunity and cancer risk, including the oncogenic impact of specific therapeutic interventions and the second, elucidating the broader systemic role of chronic inflammation. This analysis stratified data across the general population and distinct demographic groups (e.g., females, males, and elderly cohorts aged ≥66 years). Malignancy risks were isolated by organ type and further evaluated in individuals carrying a verified genetic predisposition to psoriasis, utilizing cutting-edge statistical methodologies, including one-sample, two-sample, and bidirectional MR, comprehensive meta-analyses, and PheWAS studies.

Robust causal evidence from MR, together with strong epidemiological associations from observational studies were established between psoriasis and an elevated risk of developing NMSC, mesothelioma, follicular NHL, PC, and SOS. Conversely, conflicting data and statistical inconsistencies remain regarding positive versus null associations for BC, CRC, KC, LC, and gastric cancers. Notably, no causal configurations were identified linking psoriasis to melanoma or cancers of the brain, esophagus, BLC, neck, skin, bile duct, and multiple melanoma [7,11]. Furthermore, severe statistical uncertainty persists due to wide confidence intervals regarding CNS, UDS, and EC cancers [49].

Regarding female cohorts, psoriasis demonstrated strong positive correlations with BC, anal canal cancer, NMSC, follicular NHL, and SOS, while displaying no significant associations with cervical and ovarian cancers. Interestingly, specific risks varied by drug class, as, for instance, certain registries noted a minor, standalone elevation in LC risk associated with IL-17i (HR 1.05) compared to TNF-α inhibitors [4]. On the other hand, men with an integrated genetic predisposition to psoriasis exhibited elevated risks for LC, KC, and SRDs. Geographically, tracked cohorts of diagnosed males showed strong positive associations with LC, penile, BLC, and oral cavity cancers [38]. Data for prostate cancer remain highly contradictory, with studies yielding diametrically opposed positive and negative risk indices [7,11,49]. Finally, as for geriatric cohorts (≥66 years), longitudinal data revealed a highly specific, statistically significant elevation in the incidence of anal cancer within this this subpopulation [60].

This pronounced heterogeneity stems from variations in statistical modeling, study methodologies, and population sizes. Many reviewed articles were severely restricted by small sample sizes and narrow geographic ranges (e.g., localized European cohorts) [49]. While chronic inflammation provides a biologically plausible mechanism for carcinogenesis, these studies are limited by their inability to control for unmeasured lifestyle confounders (e.g., smoking, alcohol consumption, and BMI). Moreover, several statistically significant associations reported throughout the literature were characterized by effect estimates close to unity. Although these findings may achieve statistical significance because of large sample sizes, their biological and clinical relevance remains uncertain and should therefore be interpreted alongside mechanistic evidence, consistency across study designs, and potential confounding factors [39]. Consequently, future research must utilize uniform methodologies and expansive, multi-ethnic cohorts to reduce these discrepancies and establish standard cancer screening guidelines for psoriatic patients.

### 11.2. Onco-Safety Profiles of Therapeutic Modalities

The therapeutic interventions evaluated include advanced biologicals subdivided into TNF-α, IL-12/23 (ustekinumab, guselkumab, risankizumab, and tildrakizumab), and IL-17 inhibitors, as well as non-biological therapies, including phototherapy, systemic agents like MTX, CsA, acitretin, sulfasalazine, DMF, and JAKis, and topical therapies. Due to a paucity of epidemiological data, topical treatments were analyzed from a purely theoretical perspective. Notably, broad-spectrum modalities and PUVA accelerate skin carcinogenesis, particularly in patients undergoing high cumulative session counts, carrying high risks for NMSC, BCC, SCC, and melanoma. Conversely, NB-UV_B_ represents a significantly safer profile, though rigorous long-term surveillance is mandatory [50].

MTX maintains an acceptable overall safety profile regarding generalized malignancy and specific cutaneous tumors. However, emerging registry data showing an increased risk for BCC (OR = 1.43) challenge this historical safety consensus [53]. Regarding CsA, this agent is associated with a 3.3-fold increase in relative risk for NMSC and a 1.57-fold increase in overall malignancy during long-term exposure, though short-term use appears safe [48]. It also carries elevated risks for PC (HR = 1.97) and hematological malignancies, particularly lymphoma (HR = 3.46) [47]. Concomitant or sequential deployment of CsA and PUV_A_ creates potentially synergistic increases in carcinogenic risk that requires urgent investigation [50]. Finally, both acitretin and sulfasalazine demonstrate exemplary oncological safety profiles. Acitretin does not increase malignancy risks and exerts a chemopreventive, protective effect against skin cancer when paired with phototherapy, whereas sulfasalazine similarly shows no correlation with carcinogenesis [32,48].

Advanced biological agents exhibit distinct differences in onco-safety profiles, particularly regarding lymphomagenesis. TNF-α inhibitors, and particularly continuous TNF-I therapy, is associated with significantly increased risks for total lymphoma (aHR: 2.93), NHL (aHR: 2.98), and cutaneous lymphomas (aHR: 3.46) [59]. This risk expands when tracking overall lymphoid malignancies (IRR = 1.78) and systemic lymphomas (IRR = 2.78), especially past the one-year treatment threshold. Generalized myeloid and hematological risks remain statistically non-significant [60].

IL-23 inhibitors (e.g., guselkumab) showcase an encouraging safety profile. Observed IR for NMSC track low (IR: 0.34/100 PY), while solid tumors such as BC and CRC, occur at minimal levels (IR: 0.06–0.08/100 PY) [62]. Moreover, IL-12/23 inhibitors like ustekinumab and IL-17 inhibitors confirm low malignancy risks, with NMSC IRs hovering between 0.1–0.4/100 PY [63]. While a South Korean national cohort study reported an elevated relative risk for NMSC (HR: 1.30) and general skin cancers, these findings may be restricted by ethnicity-specific factors. Additional long-term pharmacovigilance studies are required to confirm the safety of IL-17 inhibitors [47]. Direct comparisons of IL-17 and Il-23 inhibitors reveal that they offer superior oncological safety profiles over TNF-is, which have been linked to hematological malignancies during long-term use [83].

A critical consideration when interpreting the oncological safety of psoriasis therapies is the apparent paradox between suppressing chronic inflammation and inducing long-term immune modulation. Persistent systemic inflammation is considered a biologically plausible contributor of carcinogenesis through continuous cytokine signaling, oxidative stress, endothelial dysfunction, and genomic instability. Therefore, effective control of inflammation may theoretically reduce the pro-tumorigenic microenvironment associated with chronic psoriasis. Conversely, prolonged immunosuppression may impair immune surveillance, thereby reducing the host’s ability to eliminate transformed cells and potentially increasing susceptibility to selected malignancies, particularly during extended treatment or cumulative drug exposure [12,83,118].

The overall balance between these opposing mechanisms is likely to differ among therapeutic classes according to their specificity, duration of treatment, and mechanism of action. Conventional broad immunosuppressive agents such as cyclosporine and methotrexate may exert different long-term effects than targeted biological therapies directed against specific cytokine pathways. Likewise, the available evidence suggests that selective inhibition of IL-17 and IL-23 may achieve effective suppression of pathogenic inflammation, while currently demonstrating a comparatively more favorable long-term safety profile. However, current long-term evidence remains insufficient to establish direct causal relationships, and the observed associations should be interpreted cautiously considering disease severity, treatment history, cumulative exposure, and patient-related confounding factors [12,83,118].

### 11.3. Prognostic Biomarkers and Translational Insights

This review supports a biologically plausible link between chronic psoriatic inflammation and carcinogenic pathways., driven by cytokine network activation, immune dysregulation, keratinocyte hyperactivation, oxidative stress, endothelial dysfunction, and platelet-mediated inflammation. These mechanisms intersect directly with pathways governing epithelial-mesenchymal transition (EMT), tumor-promoting microenvironments, neoangiogenesis, and metastatic cascades [49,92,99,106].

Consequently, inflammatory tracking indices including NLR, PLR, SII, and SIRI serve as valuable prognostic tools. As for non-biological therapies, elevated NLR, PLR, SII, or SIRI levels correlate with significantly lower drug retention rates for MTX and CsA, indicating reduced therapeutic efficacy in highly inflammatory states [53]. Acitretin modifies select markers (NMR and PLR), but lacks long-term biomarker stability [32]. Moreover, JAKis (e.g., tofacitinib) consistently achieve high PASI_75_ responses, decrease systemic inflammatory markers, and maintain a favorable safety profile. DMF also reduced NLR values, showing early anti-inflammatory utility despite limited sample sizes [74,85].

Biological therapies, on the other hand, consistently lower systemic inflammation across NLR, PLR, SII, and CRP. TNF-α inhibitors, particularly adalimumab, provide the most stable, long-term reductions in NLR and PLR, correlating with superior clinical responses. IL-23 and IL-12/23 inhibitors, reliably reduce NLR, though their impact on PLR often lacks statistical significance [84]. IL-17 inhibitors yield variable results, as some cohorts show tight coupling between NLR reduction and PASI clearance, while others exhibit no biomarker shifting [101,102]. Overall, a reduction in NLR serves as a powerful, independent predictor of clinical treatment response.

In vivo studies and translational mouse models confirm that psoriatic inflammation extends far beyond epidermal hyperproliferation. In vivo experiments using targeted nanoparticles, such as CHALT formulations, show robust anti-inflammatory and antiproliferative activities [113]. Similarly, knockout models and selective inhibitors targeting TRPA1 ion channels and the AhR receptor demonstrate the importance of regulating cell inflammation and microvascular stability [114,117].

### 11.4. Methodological Limitations and Future Horizons

Despite these insights, several key limitations must be addressed to guide future research. First, considerable epidemiological heterogeneity, including divergent study designs, conflicting statistical methods, variable patient characteristics, restricted geographic settings, and inadequate control of confounding factors prevents a uniform consensus. Furthermore, treatment-related cancer risks are difficult to interpret because exact exposure durations, cumulative dosing metrics, and past systemic therapies are inconsistently reported across registries. Importantly, biomarker standardization is also crucial as small sample sizes, single-center designs, and unstandardized collection times limit the clinical utility of inflammatory markers like NLR and PLR [44,47,48,51,52,60,61,62,63,64,66,72,74].

To overcome these challenges, future efforts must prioritize large, multicenter longitudinal studies with standardized methodologies. Expanding research into advanced molecular targeting, novel delivery systems like nanoparticles, and mechanisms of neutrophil recruitment will pave the way for safer, targeted therapies. Given the clear links between chronic systemic inflammation, CVD events, and oncological risks, managing psoriasis effectively requires a multidisciplinary cardio-immuno-oncology approach backed by long-term patient monitoring. Further studies, integrating genomics, transcriptomics, proteomics, and artificial intelligence-driven predictive models may further improve patient stratification and facilitate personalized therapeutic strategies.

## 12. Conclusions

This review highlights the complex crossroads connecting the chronic, systemic inflammation of psoriasis to an elevated risk of specific malignancies and serious CVDs and other comorbidities. Advanced genetic and epidemiological frameworks support a distinct baseline oncological susceptibility in psoriatic patients, notably for NHL, NMSC, UDS, LC, and BC. This phenomenon is driven by overlapping, self-perpetuating cytokine networks, primarily the TNF-α, IL-23, and IL-17 axes, alongside the hyperactivation of intracellular NF-κΒ and JAK/STAT3 signaling that may promote a pro-tumorigenic microenvironment and enhance tumor cell survival.

Concurrently, anti-psoriatic therapies exhibit highly divergent safety and inflammatory profiles that require careful clinical navigation. While modern biological agents targeting the IL-17 and IL-23 pathways consistently demonstrate the most robust, sustained reductions in systemic inflammatory biomarkers (e.g., NLR) and carry a more favorable malignancy profile, conventional systemic options like CsA and certain JAKis are associated with an increased relative risk of NMSC, lymphomas, and thromboembolic events.

Overall, the relationship between psoriasis and cancer appears to be complex and multifactorial, involving both disease-related mechanisms and treatment-associated factors. Future large-scale prospective cohorts, randomized clinical studies where feasible, MR analyses, and multi-omics investigations are needed to clarify causal relationships, identify high-risk patient subgroups, validate inflammatory biomarkers, and support personalized surveillance and treatment strategies for individuals with psoriasis. Moreover, well-designed prospective multicenter studies integrating molecular biomarkers, standardized epidemiological methodologies, and long-term treatment follow-up are required before definitive causal conclusions can be established.

## Figures and Tables

**Table 1:** Summary of epidemiological evidence investigating the association between psoriasis and individual cancer types across different study designs.

Type of Cancer	Bidirectional MR	One-Sample MR	Two-Sample MR	Meta Analysis	PheWAS	Other Method	Overall Evidence	Clinical Interpretation
Colorectal cancer (CRC)	OR = 1.026 (95%, CI: 1.002–1.050)	-	OR = 1.0003	-	-	OR = 1.28 (95%, CI: 0.48–3.46)	Conflicting evidence	Mixed epidemiological evidence with no consistent causal association
Skin cancer	-	-		OR = 1.0000 (95%, CI: 0.9996–1.0005)	-	-	No convincing evidence	Genetic studies do not support a significant causal association
Melanoma Skin Cancer (MSC)	-	-	-	OR = 0.9994 (95%, CI: 0.9985–1.0002)	-	-	No convincing evidence	Genetic studies do not support a significant causal association
Non-Melanoma Skin Cancer (NMSC)	-	-	-	-	HR = 1.11 (95%, CI: 1.02–1.21)	-	Consistent positive association	Observational studies consistently report an increased risk, although causality is still uncertain
Breast cancer (BC)	OR = 0.9893 (95%, CI: 0.7619–1.2846)	OR = 1.06 (95%, CI: 1.01–1.12)	OR = 1.02 (95%, CI: 1.01–1.03)	OR = 0.9954 (95%, CI: 0.9574–1.0349)	OR = 1.01 (95%, CI: 1.00–1.01)	OR = 1.42 (95%, CI: 0.47–4.26)	Conflicting evidence	Results vary considerably among MR models and observational studies—Overall evidence remains inconclusive
Kidney cancer (KC)	-	OR = 1.25 (95%, CI: 1.09–1.42)	OR = 1.15 (95%, CI: 0.93–1.42)	OR = 1.0000 (95%, CI: 0.9998–1.0002)	OR = 1.02 (95%, CI: 1.01–1.03)	-	Conflicting evidence	Positive associations observed in some genetic analyses but are not consistently replicated in larger two-sample MR studies
Lung cancer (LC)	-	-	-	OR = 1.0003 (95%, CI: 0.9999–1.0007)	-	-	Conflicting evidence	Observational studies suggest increased risk; MR analyses do not support a causal association, indicating possible confounding by smoking and other factors
Liver cancer (LIC)	-	-	-	OR = 0.9999 (95%, CI: 0.9998–1.0000)	-	-	No convincing evidence	Current MR evidence does not support a causal association
Pancreatic cancer (PC)	-	-	-	OR = 1.0302 (95%, CI: 0.9022–1.1763)	-	-	No convincing evidence	Available MR analyses consistently show no statistically significant causal association
Central Nervous System Cancer (CNSC)	-	-	-	-	-	OR = 16.09 (95%, CI: 2.61–99.4)	Conflicting evidence	Small observational studies reported increased risk, whereas large genetic analyses found no causal association
Brain cancer	-	-	-	OR = 1.0000 (95%, CI: 1.0000–1.0002)	-	-	Strong (no association)	Current genetic evidence does not support a causal relationship
Upper Digestive System (UDS)	-	-	-	-	-	OR = 4.81 (95%, CI: 1.41–16.4)	Limited and heterogeneous evidence	Local observational studies suggest increased risk, but evidence differs considerably across individual gastrointestinal cancer types
Gastric cancer (GC)	-	OR = 0.9963 (95%, CI: 0.9724–1.0207)	-	SIR = 1.19 (95%, CI: 1.04–1.36)	-	-	Conflicting evidence	Meta-analysis suggests a modest risk increase, whereas MR studies do not support causality
Esophageal cancer	-	-	-	OR = 1.0001 (95%, CI: 0.9997–1.0003)	-	-	No convincing evidence	MR analyses consistently demonstrate no causal association
Mesothelioma	-	-	-	-	HR = 1.54 (95%, CI: 1.00–2.37)	-	Limited evidence	Observational studies suggest a positive association, but the borderline statistical significance and potential occupational confounding preclude firm conclusions
Bladder cancer (BLC)	-	-	OR = 0.999–1.0001	-	-	OR = 3.15 (95%, CI: 0.83–11.97)	Conflicting evidence	Observational studies suggest increased risk, whereas MR analyses consistently report no causal associations
Non-Hodgkin’s follicular lymphoma (NHL)	-	OR = 1.32 (95%, CI: 1.07–1.64)	-	-	OR = 1.02 (95%, CI: 1.00–1.04)	-	Consistent positive association	Multiple genetic approaches support an increased risk, making this one of the strongest associations identified in this review
Endocrine cancer (EC)	-	-	-	-	-	OR = 3.1 (95%, CI: 0.8–11.97)	Insufficient evidence	Available evidence is limited to small observational cohort with wide confidence intervals
Secondary Malignant Neoplasm of Other Sites (SOS)	-	OR = 1.10 (95%, CI: 1.03–1.17)	-	-	OR = 1.01 (95%, CI: 1.00–1.01)	-	Consistent positive association	Genetic analyses consistently demonstrate modest increases in risk, although additional validation is required

**Table 2:** Summary of current psoriasis therapies, their mechanisms of action, and the available evidence regarding malignancy risk.

Therapeutic Class	Representative Agents	Primary Molecular Target	Main Adverse Effects	Reported Malignancy Risk	Clinical Interpretation
Topical Corticosteroids	Clobetasol, Betamethasone	Suppress inflammation gene transcription	Skin atrophy, telangiectasia	No convincing long-term evidence of increased cancer risk	Favorable
Vitamin D Analogues	Calcipotriol	Regulate keratinocyte proliferation	Local irritation	No evidence of increased malignancy	Favorable
TNF-α Inhibitors	Adalimumab, Etanercept, Infliximab	TNF-α blockade	Serious infections, TB reactivation	Possible increase in lymphoma risk; overall malignancy data remain inconsistent	Moderate Concern
IL-12/23 Inhibitors	Ustekinumab	IL-12/23 blockade	Upper respiratory infections	Overall reassuring; possible small increase in skin cancer requires further study	Favorable
Il-23 Inhibitors	Guselkumab, Risankizumab, Tildrakizumab	IL-23 (*p19*) inhibition	Mild infections	No consistent increase reported	Favorable
Il-17 Inhibitors	Secukinumab, Ixekizumab, Brodalumab, Bimekizumab	Il-17 pathway inhibition	Candidiasis, neutropenia	Low incidence of malignancy in long-term studies	Favorable
Methotrexate (MTX)	-	Folate antagonist	Hepatotoxicity, myelosuppression	Conflicting evidence; possible increase in NMSC and lymphoma	Moderate concern
Cyclosporine (CsA)	-	Calcineurin inhibition	Nephrotoxicity, hypertension	Increased risk with prolonged exposure, mainly skin cancer	Higher concern
Acitretin	-	Retinoid	Teratogenicity, mucocutaneous effects	No consistent increase; may reduce SCC risk	Favorable
JAK Inhibitors	Tofacitinib, Deucravacitinib	JAK-STAT pathway inhibition	Herpes zoster, infections	Higher concern for lymphoma and NMSC compared to other biologics	Requires careful monitoring

**Table 3:** Effects of Psoriasis Therapies on Inflammatory Biomarkers, Implications and Clinical Outcomes.

Therapeutic Class		Representative Agents	Main Inflammatory Biomarkers Affected	Effects on Psoriasis Activity	Clinical Implication
Conventional Systemic Therapies	Methotrexate (MTX)	-	Platelets, PLR, SII	High baseline inflammatory markers predict poor drug survival	Baseline inflammation predicts treatment persistence
	Cyclosporine (CsA)	-	Platelets, Neutrophils, PLR, SII	Similar profile to MTX	Reduced drug survival with high inflammatory burden
	Acitretin	-	NMR, PLR	Temporary reductions	Limited sustained effect
Targeted Synthetic Therapies	JAK Inhibitors (JAKis)	Tofacitinib, Baricitinib, etc.	Limited evidence	High PASI scores	Effective systemic therapy
	Dimethyl Fumarate (DMF)	-	NLR	Reduced NLR	Possible systemic anti-inflammatory effect
Biological Treatments	TNF-α Inhibitors (TNFis)	Adalimumab, Infliximab, Etanercept	NLR, PLR, IL-6, SII, CRP	Consistent reductions	Biomarker improvement parallels PASI improvement
	IL-23 Inhibitors	Guselkumab, Risankizumab, Tildrakizumab	NLR, PLR	Reduced NLR	Excellent drug survival
	IL-12/23 Inhibitors	Ustekinumab	NLR, PLR	Reduced NLR and PLR	Sustained efficacy
	IL-17 Inhibitors	Secukinumab, Ixekizumab, Brodalumab	Mainly NLR	NLR decreases during treatment	Good biomarker for monitoring

**Table 4:** Effects of psoriasis therapies on inflammatory biomarkers, clinical outcomes, and cardiovascular implications.

Therapy/Intervention		Cardiovascular Effect	Main Findings	Clinical Interpretation
Anti-Psoriatic Therapies	JAK Inhibitors (JAKis)	Increased thromboembolic risk	Increased PE; careful monitoring	Use cautiously in high-risk patients
	Systemic Corticosteroids	Increased CVD risk	Increased DVT, PE, and MI risk	Avoid chronic use
	TNF-α Inhibitors (TNFis)	Mixed observations	Mostly neutral and protective; Adalimumab showed safety signals	Individualized assessment
Adjunctive and Non-Anti-Psoriatic Therapies	Statins	Cardioprotective	Improved lipid metabolism and inflammatory profile	Beneficial adjunctive therapy
	Aspirin	Reduced platelet activation	Reduced T_X_B_2_ and endothelial inflammation	Potential vascular protection
	Systemic/Biologic Therapies	Improved vascular biomarkers	RDW normalization in responders	May reflect reduced CVD risk
	Sphingosine 1-phosphate (S1P) Modulators	Possible beneficial outcomes	Reduced ischemic stroke and possible vascular benefit	Emerging evidence
	Hormone Replacement Therapy (HRT)	Reduced DVT/PE/stroke	Significant reductions in CVDs	Not psoriasis-specific
	Oral Contraceptives	Increased DVT	Limited evidence	Consider individual risk

**Table 5:** Comparative effects of different treatment categories on inflammatory markers and clinical response in psoriasis.

Treatment Category	Agent	Influenced Inflammatory Markers *	Statistically Significant Changes	Clinical Response
TNF-α Inhibitors	Adalimumab	d-NLR ↓	0.014	Moderate
	Etanercept	SII ↓, SIRI ↓	0.04, 0.01	Moderate
	Certolizumab	AISI ↓	0.04	Limited data
IL-17 Inhibitors	Ixekizumab	NLR ↓, SIRI ↓	0.04, 0.008	High
	Secukinumab	NLR ↓, MLR ↓, AISI ↓	0.005, 0.008, 0.008	High
IL-23 Inhibitors	Risankizumab	NLR ↓	0.04	High
	Guselkumab	NLR ↓, SII ↓	0.02, 0.04	Moderate
Non-Biological	Apremilast	No significant changes	>0.05	Low [83]

* ↓: Marker downregulation.

**Table 6:** Comparison of biologic treatment groups according to changes in inflammatory markers in psoriasis.

Treatment Group	Agents	Patients (n/%)	Inflammatory Markers Evaluated	Statistical Significance (*p*-Value)	Overall Direction of Change
Anti-TNF-α	Adalimumab	7 (12.72%)	Neutrophils, Platelets, NLR, PLR, PCT, CRP	*p* = 0.008, 0.012, 0.017, 0.001, 0.011, 0.009	Significant decrease
	Certolizumab				
Anti-IL-12/23	Ustekinumab	9 (16.36%)		*p* = 0.008, 0.012, 0.017, 0.001, 0.011, 0.009	Significant decrease
Anti-IL-17	Secukinumab	34 (61.82%)		*p* = 0.008, 0.012, 0.017, 0.001, 0.011, 0.009	Significant decrease
	Ixekizumab				
Anti-IL-23	Guselkumab	5 (9.1%)		*p* = 0.008, 0.012, 0.017, 0.001, 0.011, 0.009	Significant decrease
	Risankizumab				

**Table 7:** Comparison of the malignancy risk associated with JAK inhibitors versus TNF-α inhibitors in psoriasis.

Cancer Type	Treatment Class	Agents	Effect Estimate
Lymphoma (Hodgkin and non-Hodgkin (NHL))	JAK Inhibitors	Tofacitinib Baricitinib, Upadacitinib	IRR (JAKis vs. TNFis) = 1.48
	TNF-α Inhibitors	Adalimumab, Etanercept, Infliximab	
Non-melanoma skin cancer (NMSC)	JAK Inhibitors	Tofacitinib	IRR (JAKis vs. TNFis) = 2.12 (95%, CI: 1.32–3.41)
	TNF-α Inhibitors	Adalimumab, Etanercept, Infliximab	
Lung cancer (LC)	JAK Inhibitors	Tofacitinib	RR (JAKis vs. TNFis) = 1.82
	TNF-α Inhibitors	Adalimumab, Etanercept, Infliximab	
Overall cancer risk (including NMSC)	JAK Inhibitors	All JAK inhibitors	IRR (JAKis vs. TNFis) = 1.50 (95%, CI: 1.16–1.94)
	TNF-α Inhibitors	All TNF-α inhibitors	

**Table 8:** Comparison of the malignancy risk associated with IL-17 and IL-23 inhibitors versus TNF-α inhibitors in patients with psoriasis.

Cancer Type	IL-17 Inhibitors vs. TNFis	IL-23 Inhibitors vs. TNFis
Non-Hodgkin lymphoma (NHL)	Reduced risk (HR 0.58)	Reduced risk (HR = 0.39)
Colorectal cancer (CRC)	Reduced risk (HR 0.68)	No significant association
Hepatobiliary cancer	Reduced risk (HR 0.68)	Reduced risk (HR = 0.44)
Ovarian cancer	Reduced risk (HR 0.48)	No significant association
Melanoma	Reduced risk (HR 0.52)	No significant association
Basal cell carcinoma	Reduced risk (HR 0.57)	Reduced risk (HR = 0.76)

## Data Availability

No new data were created or analyzed in this study. Data sharing is not applicable to this article.

## References

[1] Wei Q., Wang Z., Liu X., Liang H., Chen L. (2023). Association between Gastric Cancer and 12 Autoimmune Diseases: A Mendelian Randomization Study. Genes.

[2] Chen L., Wang F., Zhang H., Cao B. (2024). Exploring Potential Causal Associations between Autoimmune Diseases and Colorectal Cancer Using Bidirectional Mendelian Randomization. Sci. Rep..

[3] Mörth C., Valachis A., Abu Sabaa A., Marshall K., Hedström G., Flogegård M., Baecklund E., Enblad G. (2019). Autoimmune Disease in Patients with Diffuse Large B-Cell Lymphoma: Occurrence and Impact on Outcome. Acta Oncol..

[4] Pillai A., Valero C., Zanoni D., Navas K., Morris Q., Ganly I., Patel S.G. (2022). Prognostic Impact of Autoimmune Disease in Oral Cavity Squamous Cell Carcinoma. J. Surg. Oncol..

[5] Song X., Liang H., Nan F., Chen W., Li J., He L., Cun Y., Li Z., Zhang W., Zhang D. (2025). Autoimmune Diseases: Molecular Pathogenesis and Therapeutic Targets. MedComm.

[6] Trovato E., Dragotto M., Capalbo E., Cartocci A., Rubegni P., Calabrese L. (2024). Risk of Skin Cancer in Patients with Psoriasis: Single-Center Retrospective Study Comparing Anti-TNFα and Phototherapy. J. Clin. Med..

[7] Long J., Yang M., Pang Y., Kang H., Liang S., Wang D. (2024). The Causal Relationship between Psoriasis and Cancers: A Two-Sample Mendelian Randomization Analysis. Front. Oncol..

[8] Adil S., Paracha R.Z., Tariq S., Nisar M., Ijaz S., Siddiqa A., Hussain Z., Amir A. (2021). A Computational Systems Analyses to Identify Biomarkers and Mechanistic Link in Psoriasis and Cutaneous Squamous Cell Carcinoma. Front. Immunol..

[9] Fung K.Y., Louis C., Metcalfe R.D., Kosasih C.C., Wicks I.P., Griffin M.D.W., Putoczki T.L. (2022). Emerging Roles for IL-11 in Inflammatory Diseases. Cytokine.

[10] Kim S.Y., Yoo D.M., Chung J., Choi H.G. (2022). Thyroid Cancer and Psoriasis: A Nested Case–Control Study. Diagnostics.

[11] Gheucă-Solovăstru L., Vâţă D., Halip A.I., Patraşcu A., Cozma A., Porumb-Andrese E. (2021). Psoriasis—A Cancer Risk Factor?. Appl. Sci..

[12] Tang L., Yang X., Liang Y., Xie H., Dai Z., Zheng G. (2018). Transcription Factor Retinoid-Related Orphan Receptor Γt: A Promising Target for the Treatment of Psoriasis. Front. Immunol..

[13] Güven A.T., Şener Y.Z., Özdede M. (2023). Psoriatic Inflammation-Induced Atypically Located Venous Thromboembolism: A Case of Immuno-Thrombosis. Niger. J. Clin. Pract..

[14] Arachchillage D.J., Mobayen G., Laffan M., Randi A.M., Ahnström J., Pericleous C. (2025). A Cell-Based Model to Study Mechanisms of Endothelial-Dependent Thrombin Generation in Response to Inflammation and Its Modulation by Hydroxychloroquine. Res. Pract. Thromb. Haemost..

[15] Grover S.P., Mackman N. (2018). Tissue Factor: An Essential Mediator of Hemostasis and Trigger of Thrombosis. Arter. Thromb. Vasc. Biol..

[16] Mir M.M., Dogra D., Koul K.K. (2022). Hemostatic and Coagulation Profile in Psoriasis: A Hospital-Based Case Control Study. Indian J. Dermatol..

[17] Ogdie A., Kay McGill N., Shin D.B., Takeshita J., Jon Love T., Noe M.H., Chiesa Fuxench Z.C., Choi H.K., Mehta N.N., Gelfand J.M. (2018). Risk of Venous Thromboembolism in Patients with Psoriatic Arthritis, Psoriasis and Rheumatoid Arthritis: A General Population-Based Cohort Study. Eur. Heart J..

[18] Brandum E.P., Jørgensen A.S., Rosenkilde M.M., Hjortø G.M. (2021). Dendritic Cells and CCR7 Expression: An Important Factor for Autoimmune Diseases, Chronic Inflammation, and Cancer. Int. J. Mol. Sci..

[19] Dincer Rota D., Tanacan E. (2021). The Utility of Systemic-Immune Inflammation Index for Predicting the Disease Activation in Patients with Psoriasis. Int. J. Clin. Pract..

[20] Wang W.-M., Wu C., Gao Y.-M., Li F., Yu X.-L., Jin H.-Z. (2021). Neutrophil to Lymphocyte Ratio, Platelet to Lymphocyte Ratio, and Other Hematological Parameters in Psoriasis Patients. BMC Immunol..

[21] Li L., Yao D., Lu Y., Deng J., Wei J., Yan Y., Deng H., Han L., Lu C. (2020). Metabonomics Study on Serum Characteristic Metabolites of Psoriasis Vulgaris Patients with Blood-Stasis Syndrome. Front. Pharmacol..

[22] Solak B., Kara R.Ö. (2024). Assessing Systemic Inflammatory Markers in Psoriasis: A Retrospective Study. Trop. Med. Int. Health.

[23] Nowowiejska J., Baran A., Hermanowicz J.M., Sieklucka B., Pawlak D., Flisiak I. (2023). Evaluation of Plasma Concentrations of Galectins-1, 2 and 12 in Psoriasis and Their Clinical Implications. Biomolecules.

[24] Zhao X., Li J., Li X. (2024). Association between Systemic Immune-Inflammation Index and Psoriasis: A Population-Based Study. Front. Immunol..

[25] Guo H., Chen R. (2024). Association of Systemic Inflammation Index with Psoriasis Risk and Psoriasis Severity: A Retrospective Cohort Study of NHANES 2009 to 2014. Medicine.

[26] Ma R., Cui L., Cai J., Yang N., Wang Y., Chen Q., Chen W., Peng C., Qin H., Ding Y. (2024). Association between Systemic Immune Inflammation Index, Systemic Inflammation Response Index and Adult Psoriasis: Evidence from NHANES. Front. Immunol..

[27] Mondal S., Guha S., Saha A., Ghoshal L., Bandyopadhyay D. (2022). Evaluation of Neutrophil-to-Lymphocyte Ratio and Platelet-to-Lymphocyte Ratio in Chronic Plaque Psoriasis. Indian J. Dermatol..

[28] Sirin M.C., Korkmaz S., Erturan I., Filiz B., Aridogan B.C., Cetin E.S., Yildirim M. (2020). Evaluation of Monocyte to HDL Cholesterol Ratio and Other Inflammatory Markers in Patients with Psoriasis. An. Bras. Dermatol..

[29] Lewandowska J.A., Owczarczyk–Saczonek A. (2026). The Role of Inflammatory Biomarkers in Disease Severity and Treatment Response across Psoriasis, Atopic Dermatitis, and Hidradenitis Suppurativa—A Narrative Review. Dermatol. Ther..

[30] Zhang Y., Qian H., Kuang Y.-H., Wang Y., Chen W.-Q., Zhu W. (2024). Evaluation of the Inflammatory Parameters as Potential Biomarkers of Systemic Inflammation Extent and the Disease Severity in Psoriasis Patients. Arch. Dermatol. Res..

[31] Tiucă O.M., Morariu S.H., Mariean C.R., Tiucă R.A., Nicolescu A.C., Cotoi O.S. (2024). Impact of Blood-Count-Derived Inflammatory Markers in Psoriatic Disease Progression. Life.

[32] Kiluk P., Baran A., Kaminski T.W., Maciaszek M., Flisiak I. (2019). The Level of FGF 21 as a New Risk Factor for the Occurrence of Cardiometabolic Disorders amongst the Psoriatic Patients. J. Clin. Med..

[33] Conic R.R., Damiani G., Schrom K.P., Ramser A.E., Zheng C., Xu R., McCormick T.S., Cooper K.D. (2020). Psoriasis and Psoriatic Arthritis Cardiovascular Disease Endotypes Identified by Red Blood Cell Distribution Width and Mean Platelet Volume. J. Clin. Med..

[34] Garshick M.S., Block R., Drenkova K., Tawil M., James G., Brenna J.T. (2022). Statin Therapy Upregulates Arachidonic Acid Status via Enhanced Endogenous Synthesis in Patients with Plaque Psoriasis. Prostaglandins Leukot. Essent. Fat. Acids.

[35] Garshick M.S., Tawil M., Barrett T.J., Salud-Gnilo C.M., Eppler M., Lee A., Scher J.U., Neimann A.L., Jelic S., Mehta N.N. (2020). Activated Platelets Induce Endothelial Cell Inflammatory Response in Psoriasis via COX-1. Arterioscler. Thromb. Vasc. Biol..

[36] Song M., Engels E.A., Clarke M.A., Kreimer A.R., Shiels M.S. (2024). Autoimmune Disease and the Risk of Anal Cancer in the US Population Aged 66 Years and Over. JNCI J. Natl. Cancer Inst..

[37] Fischer S., Meisinger C., Freuer D. (2024). Autoimmune Diseases and Female-Specific Cancer Risk: A Systematic Review and Meta-Analysis. J. Autoimmun..

[38] Li R., Luo W., Chen X., Zeng Q., Yang S., Wang P., Hu J., Chen A. (2024). An Observational and Genetic Investigation into the Association between Psoriasis and Risk of Malignancy. Nat. Commun..

[39] Clinical Chemistry—Edition 9—By William J. Marshall, MA, PhD, MSc, MBBS, FRCP, FRCPath, FRCPEdin, FRSB, FRSC, Márta Lapsley, MB BCh BAO, MD, FRCPath, Andrew Day, MA MSc MBBS FRCPath and Kate ShipmanElsevier Health Inspection Copies. http://educate.elsevier.com/book/details/9780702079368.

[40] Zádori N., Szakó L., Váncsa S., Vörhendi N., Oštarijaš E., Kiss S., Frim L., Hegyi P., Czimmer J. (2021). Six Autoimmune Disorders Are Associated with Increased Incidence of Gastric Cancer: A Systematic Review and Meta-Analysis of Half a Million Patients. Front. Immunol..

[41] Sauter J.L., Dacic S., Galateau-Salle F., Attanoos R.L., Butnor K.J., Churg A., Husain A.N., Kadota K., Khoor A., Nicholson A.G. (2022). The 2021 WHO Classification of Tumors of the Pleura: Advances Since the 2015 Classification. J. Thorac. Oncol..

[42] Trafford A.M., Parisi R., Kontopantelis E., Griffiths C.E.M., Ashcroft D.M. (2019). Association of Psoriasis with the Risk of Developing or Dying of Cancer. JAMA Dermatol..

[43] Sarabia S., Ranjith B., Koppikar S., Wijeratne D.T. (2022). Efficacy and Safety of JAK Inhibitors in the Treatment of Psoriasis and Psoriatic Arthritis: A Systematic Review and Meta-Analysis. BMC Rheumatol..

[44] Sugimoto E., Matsuda H., Shibata S., Mizuno Y., Koyama A., Li L., Taira H., Ito Y., Awaji K., Yamashita T. (2023). Impact of Pretreatment Systemic Inflammatory Markers on Treatment Persistence with Biologics and Conventional Systemic Therapy: A Retrospective Study of Patients with Psoriasis Vulgaris and Psoriatic Arthritis. J. Clin. Med..

[45] Esse S., Mason K.J., Green A.C., Warren R.B. (2020). Melanoma Risk in Patients Treated With Biologic Therapy for Common Inflammatory Diseases: A Systematic Review and Meta-Analysis. JAMA Dermatol..

[46] Andersen C.S.B., Kvist-Hansen A., Siewertsen M., Enevold C., Hansen P.R., Kaur-Knudsen D., Zachariae C., Nielsen C.H., Loft N., Skov L. (2023). Blood Cell Biomarkers of Inflammation and Cytokine Levels as Predictors of Response to Biologics in Patients with Psoriasis. Int. J. Mol. Sci..

[47] Hong J.Y., Ahn J., Won S., Kim S.M., Cho Y.A., Kim C.Y., Sung J.Y., Yu D.-A., Lee Y.W., Choe Y.B. (2022). Risk of Malignancy in Patients with Psoriasis According to Treatment Modalities in Korea: A Nationwide Cohort Study. Sci. Rep..

[48] Chou Y.-J., Wu C.-Y., Pan T.-Y., Wu C.-Y., Chang Y.-T. (2022). Risk of Malignancy in Patients with Psoriasis Receiving Systemic Medications: A Nested Case-Control Study. Dermatol. Ther..

[49] Sevdem G.A., Ozlem K.S., Nazan Y., Nahide O. (2024). Does the Risk of Basal Cell Carcinoma Increase in Patients with Psoriasis under Treatment?. Photodermatol. Photoimmunol. Photomed..

[50] Akyol M. (2022). Phototherapy. Turkderm.

[51] Giannopoulos F., Gillstedt M., Laskowski M., Kristensen K.B., Polesie S. (2021). Methotrexate Use for Patients with Psoriasis and Risk of Cutaneous Squamous Cell Carcinoma: A Nested Case-Control Study. Acta Derm.-Venereol..

[52] Cai S., Perng W.-T., Huang J.Y., Chiou J.-Y., Dong L., Wei J.C. (2020). Neoplasm Risk in Rheumatic Diseases Has No Correlation with Conventional Synthetic Disease-Modifying Anti-Rheumatic Drugs Usage—A Population-Based Nested Case–Control Study. Front. Med..

[53] Polesie S., Gillstedt M., Schmidt S.A.J., Egeberg A., Pottegård A., Kristensen K. (2023). Use of Methotrexate and Risk of Skin Cancer: A Nationwide Case–Control Study. Br. J. Cancer.

[54] Xue C., Yao Q., Gu X., Shi Q., Yuan X., Chu Q., Bao Z., Lu J., Li L. (2023). Evolving Cognition of the JAK-STAT Signaling Pathway: Autoimmune Disorders and Cancer. Sig. Transduct. Target. Ther..

[55] Russell M.D., Stovin C., Alveyn E., Adeyemi O., Chan C.K.D., Patel V., Adas M.A., Atzeni F., Ng K.K.H., Rutherford A.I. (2023). JAK Inhibitors and the Risk of Malignancy: A Meta-Analysis across Disease Indications. Ann. Rheum. Dis..

[56] Mammoliti O., Menet C., Cottereaux C., Blanc J., De Blieck A., Coti G., Geney R., Oste L., Ostyn K., Palisse A. (2024). Design of a Potent and Selective Dual JAK1/TYK2 Inhibitor. Bioorg. Med. Chem..

[57] Bao Y., Xu R., Guo J. (2024). The Multiple-Action Allosteric Inhibition of TYK2 by Deucravacitinib: Insights from Computational Simulations. Comput. Biol. Chem..

[58] Lee H.-J., Kim M. (2023). Challenges and Future Trends in the Treatment of Psoriasis. Int. J. Mol. Sci..

[59] Kim H.W., Kim E.H., Lee M., Jung I., Ahn S.S. (2023). Risk of Cancer, Tuberculosis and Serious Infections in Patients with Ankylosing Spondylitis, Psoriatic Arthritis and Psoriasis Treated with IL-17 and TNF-α Inhibitors: A Nationwide Nested Case-Control Analysis. Clin. Exp. Rheumatol..

[60] Tsai C.-J., Lin Y.-C., Chen C.-Y., Hung C.-H., Lin Y.-C. (2023). The Effects of Biologics on Hematologic Malignancy Development in Patients with Ankylosing Spondylitis, Psoriasis, or Psoriatic Arthritis: A National Cohort Study. Biomedicines.

[61] Jung J.M., Kim Y.-J., Chang S.E., Lee M.W., Won C.H., Lee W.J. (2023). The Risk of Lymphoproliferative Disorders and Skin Cancers in Patients with Psoriasis and Inflammatory Bowel Disease Administered Biologics. Dermatol. Ther..

[62] Blauvelt A., Lebwohl M., Langley R.G., Rowland K., Yang Y.-W., Chan D., Miller M., You Y., Yu J., Thaҫi D. (2023). Malignancy Rates through 5 Years of Follow-up in Patients with Moderate-to-Severe Psoriasis Treated with Guselkumab: Pooled Results from the VOYAGE 1 and VOYAGE 2 Trials. J. Am. Acad. Dermatol..

[63] Gottlieb A., Lebwohl M., Liu C., Israel R.J., Jacobson A. (2020). Malignancy Rates in Brodalumab Clinical Studies for Psoriasis. Am. J. Clin. Dermatol..

[64] Lebwohl M.G., Koo J.Y., Armstrong A.W., Strober B.E., Martin G.M., Rawnsley N.N., Goehring E.L., Jacobson A.A. (2024). Brodalumab: 5-Year US Pharmacovigilance Report. Dermatol. Ther..

[65] Smith S.D., Stratigos A., Augustin M., Carrascosa J.M., Grond S., Riedl E., Xu W., Patel H., Lebwohl M. (2023). Integrated Safety Analysis on Skin Cancers among Patients with Psoriasis Receiving Ixekizumab in Clinical Trials. Dermatol. Ther..

[66] Albayrak H. (2023). Neutrophil-to-Lymphocyte Ratio, Neutrophil-to-Monocyte Ratio, Platelet-to-Lymphocyte Ratio, and Systemic Immune-Inflammation Index in Psoriasis Patients: Response to Treatment with Biological Drugs. J. Clin. Med..

[67] Şentürk N. (2022). Tofacitinib. Turkderm.

[68] Kerschbaumer A., Smolen J.S., Nash P., Doerner T., Dougados M., Fleischmann R., Geissler K., McInnes I.B., Takeuchi T., Trauner M. (2020). Points to Consider for the Treatment of Immune-Mediated Inflammatory Diseases with Janus Kinase Inhibitors: A Systematic Literature Research. RMD Open.

[69] Kommoss K.S., Bieler T., Ringen J., Lehmann A., Mihalceanu S., Hobohm L., Keller K., Brand A., Fischer B., Kramer D. (2024). A Simple Tool for Evaluation of Inflammation in Psoriasis: Neutrophil-to-Lymphocyte and Platelet-to-Lymphocyte Ratio as Markers in Psoriasis Patients and Related Murine Models of Psoriasis-like Skin Disease. J. Mol. Med..

[70] An I., Ucmak D., Ozturk M. (2020). The Effect of Biological Agent Treatment on Neutrophil-to-Lymphocyte Ratio, Platelet-to-Lymphocyte Ratio, Mean Platelet Volume, and C-Reactive Protein in Psoriasis Patients. Adv. Dermatol. Allergol..

[71] Najar Nobari N., Shahidi Dadras M., Nasiri S., Abdollahimajd F., Gheisari M. (2020). Neutrophil/Platelet to Lymphocyte Ratio in Monitoring of Response to TNF-α Inhibitors in Psoriatic Patients. Dermatol. Ther..

[72] Warren R.B., Marsden A., Tomenson B., Mason K.J., Soliman M.M., Burden A.D., Reynolds N.J., Stocken D., Emsley R., Griffiths C.E.M. (2019). Identifying Demographic, Social and Clinical Predictors of Biologic Therapy Effectiveness in Psoriasis: A Multicentre Longitudinal Cohort Study. Br. J. Dermatol..

[73] Eyerich K., Asadullah K., Pinter A., Weisenseel P., Reich K., Paul C., Sabat R., Wolk K., Eyerich S., Lauffer F. (2024). Noninferiority of 16-Week vs 8-Week Guselkumab Dosing in Super Responders for Maintaining Control of Psoriasis: The GUIDE Randomized Clinical Trial. JAMA Dermatol..

[74] Taşkın B., Memet B., Vurgun E., Alper S. (2021). Decrease in Inflammation Markers with Ustekinumab Treatment in Moderate-Severe Psoriasis. Pam. Med. J..

[75] An İ., Aksoy M., Ayhan E., Ozturk M. (2021). The Effect of Secukinumab Treatment on Inflammatory Parameters in Patients with Psoriasis: A Multicentre Restrospective Study. Int. J. Clin. Pract..

[76] Setyawan J., Mu F., Yarur A., Zichlin M.L., Yang H., Fernan C., Billmyer E., Downes N., Azimi N., Strand V. (2021). Risk of Thromboembolic Events and Associated Risk Factors, Including Treatments, in Patients with Immune-Mediated Diseases. Clin. Ther..

[77] Mease P., Charles-Schoeman C., Cohen S., Fallon L., Woolcott J., Yun H., Kremer J., Greenberg J., Malley W., Onofrei A. (2020). Incidence of Venous and Arterial Thromboembolic Events Reported in the Tofacitinib Rheumatoid Arthritis, Psoriasis and Psoriatic Arthritis Development Programmes and from Real-World Data. Ann. Rheum. Dis..

[78] Ma J., Cai J., Chen H., Feng Z., Yang G. (2024). Cardiovascular Adverse Events Associated with Tumor Necrosis Factor-Alpha Inhibitors: A Real-World Pharmacovigilance Analysis. J. Atheroscler. Thromb..

[79] Nguyen T.T., Jensen C.G., Khoury L., Deleuran B., Blom E.S., Breinholt T., Christensen R., Skov L. (2021). Effectiveness of Mind–Body Intervention for Inflammatory Conditions: Results from a 26-Week Randomized, Non-Blinded, Parallel-Group Trial. J. Clin. Med..

[80] Grine L., Hilhorst N., Michels N., Abbeddou S., Henauw S.D., Lambert J. (2022). The Effects of Modified Intermittent Fasting in Psoriasis (MANGO): Protocol for a Two-Arm Pilot Randomized Controlled Open Cross-over Study. JMIR Res. Protoc..

[81] Meneguin S., de Godoy N.A., Pollo C.F., Miot H.A., de Oliveira C. (2020). Quality of Life of Patients Living with Psoriasis: A Qualitative Study. BMC Dermatol..

[82] Blake T., Gullick N.J., Hutchinson C.E., Bhalerao A., Wayte S., Weedall A., Barber T.M. (2023). More than Skin-Deep: Visceral Fat Is Strongly Associated with Disease Activity, Function and Metabolic Indices in Psoriatic Disease. Arthritis Res. Ther..

[83] Morariu S.-H., Cotoi O.S., Tiucă O.M., Baican A., Gheucă-Solovăstru L., Decean H., Brihan I., Silaghi K., Biro V., Șerban-Pescar D. (2024). Blood-Count-Derived Inflammatory Markers as Predictors of Response to Biologics and Small-Molecule Inhibitors in Psoriasis: A Multicenter Study. J. Clin. Med..

[84] Kulaklı S., Oğuz I. (2023). Effect of Biological Therapy on Systemic Inflammatory Markers Among Patients with Chronic Plaque Psoriasis. Med. Bull. Haseki.

[85] Saeki H., Mabuchi T., Asahina A., Abe M., Igarashi A., Imafuku S., Okubo Y., Komine M., Takahashi K., Torii H. (2023). English Version of Japanese Guidance for the Use of Oral Janus Kinase Inhibitors (JAK1 and TYK2 Inhibitors) in the Treatments of Psoriasis. J. Dermatol..

[86] Kridin K., Abdelghaffar M., Mruwat N., Ludwig R.J., Thaçi D. (2024). Are Interleukin 17 and Interleukin 23 Inhibitors Associated with Malignancies?—Insights from an International Population-Based Study. J. Eur. Acad. Dermatol. Venereol..

[87] Guo J., Zhang H., Lin W., Lu L., Su J., Chen X. (2023). Signaling Pathways and Targeted Therapies for Psoriasis. Sig. Transduct. Target. Ther..

[88] Sieminska I., Pieniawska M., Grzywa T.M. (2024). The Immunology of Psoriasis-Current Concepts in Pathogenesis. Clin. Rev. Allergy Immunol..

[89] Man A.-M., Orăsan M.S., Hoteiuc O.-A., Olănescu-Vaida-Voevod M.-C., Mocan T. (2023). Inflammation and Psoriasis: A Comprehensive Review. Int. J. Mol. Sci..

[90] Wang L., Liu R., Tang Y., Ma Y., Wang G., Ruan Q., Zheng S.J. (2025). Advances in Psoriasis Research: Decoding Immune Circuits and Developing Novel Therapies. Int. J. Mol. Sci..

[91] Bruni M., Lobefaro F., Pellegrini C., Mastrangelo M., Gualdi G., Esposito M., Antonetti P., De Sanctis P., Amerio P., Fargnoli M.C. (2025). Psoriasis and Cancer: The Role of Inflammation, Immunosuppression, and Cancer Treatment. Expert Opin. Biol. Ther..

[92] Giraulo C., De Palma G., Plaitano P., Esposito R., Morretta E., Monti M.C., Müller C.E., Cicala C., Morello S. (2026). Oncostatin M Upregulates CD73 via the MAPK Pathway in Keratinocytes to Promote an Adenosine-Dependent Anti-Inflammatory Response in Psoriasis. Front. Immunol..

[93] Kim J., Moreno A., Krueger J.G. (2022). The Imbalance between Type 17 T-Cells and Regulatory Immune Cell Subsets in Psoriasis Vulgaris. Front. Immunol..

[94] Jang D., Lee A.-H., Shin H.-Y., Song H.-R., Park J.-H., Kang T.-B., Lee S.-R., Yang S.-H. (2021). The Role of Tumor Necrosis Factor Alpha (TNF-α) in Autoimmune Disease and Current TNF-α Inhibitors in Therapeutics. Int. J. Mol. Sci..

[95] Bai W., Wang K., Wu S., Zhou X., Liu L., Liu M., Chen C. (2025). Identification of STAT3 and BIRC5 as Anoikis-Related Biomarkers in Psoriasis. Sci. Rep..

[96] Liu T., Li S., Ying S., Tang S., Ding Y., Li Y., Qiao J., Fang H. (2020). The IL-23/IL-17 Pathway in Inflammatory Skin Diseases: From Bench to Bedside. Front. Immunol..

[97] Rani H., Saini N. (2025). Overexpression of miR-718 in Keratinocytes Affects the Psoriatic Inflammation via Targeting STAT1: In Vitro and In Vivo Evidence. Clin. Exp. Pharmacol. Physiol..

[98] Huang B., Lang X., Li X. (2022). The Role of IL-6/JAK2/STAT3 Signaling Pathway in Cancers. Front. Oncol..

[99] Wen Y., Zhu Y., Zhang C., Yang X., Gao Y., Li M., Yang H., Liu T., Tang H. (2022). Chronic Inflammation, Cancer Development and Immunotherapy. Front. Pharmacol..

[100] Dobrică E.-C., Cozma M.-A., Găman M.-A., Voiculescu V.-M., Găman A.M. (2022). The Involvement of Oxidative Stress in Psoriasis: A Systematic Review. Antioxidants.

[101] Xu F., Xu J., Xiong X., Deng Y. (2019). Salidroside Inhibits MAPK, NF-κB, and STAT3 Pathways in Psoriasis-Associated Oxidative Stress via SIRT1 Activation. Redox. Rep..

[102] Pleńkowska J., Gabig-Cimińska M., Mozolewski P. (2020). Oxidative Stress as an Important Contributor to the Pathogenesis of Psoriasis. Int. J. Mol. Sci..

[103] Andrés R.M., Montesinos M.C., Navalón P., Payá M., Terencio M.C. (2013). NF-κB and STAT3 Inhibition as a Therapeutic Strategy in Psoriasis: In Vitro and In Vivo Effects of BTH. J. Investig. Dermatol..

[104] Zhou X., Chen Y., Cui L., Shi Y., Guo C. (2022). Advances in the Pathogenesis of Psoriasis: From Keratinocyte Perspective. Cell Death Dis..

[105] Shou Y., Yang L., Yang Y., Xu J. (2021). Inhibition of Keratinocyte Ferroptosis Suppresses Psoriatic Inflammation. Cell Death Dis..

[106] Liang N., Zhang K. (2026). Factors Influencing the Proliferation of Keratinocytes in Psoriasis. iScience.

[107] Li L., Liu J., Lu J., Wu J., Zhang X., Ma T., Wu X., Zhu Q., Chen Z., Tai Z. (2025). Interventions in Cytokine Signaling: Novel Horizons for Psoriasis Treatment. Front. Immunol..

[108] Zwain A., Aldiwani M., Taqi H. (2021). The Association Between Psoriasis and Cardiovascular Diseases. Eur. Cardiol..

[109] Jiang Z., Jiang X., Chen A., He W. (2023). Platelet Activation: A Promoter for Psoriasis and Its Comorbidity, Cardiovascular Disease. Front. Immunol..

[110] Tsoupras A., Adamantidi T., Finos M.A., Philippopoulos A., Detopoulou P., Tsopoki I., Kynatidou M., Demopoulos C.A., Tsoupras A., Adamantidi T. (2024). Re-Assessing the Role of Platelet Activating Factor and Its Inflammatory Signaling and Inhibitors in Cancer and Anti-Cancer Strategies. Front. Biosci..

[111] Tsoupras A.B., Iatrou C., Frangia C., Demopoulos C.A. (2009). The Implication of Platelet Activating Factor in Cancer Growth and Metastasis: Potent Beneficial Role of PAF-Inhibitors and Antioxidants. Infect. Disord. Drug. Targets.

[112] Salamah M.F., Vallance T.M., Kodji X., Ravishankar D., Williams H.F., Brain S.D., Vaiyapuri S. (2020). The Antimicrobial Cathelicidin CRAMP Augments Platelet Activation during Psoriasis in Mice. Biomolecules.

[113] Chen R., Zhai Y.-Y., Sun L., Wang Z., Xia X., Yao Q., Kou L. (2022). Alantolactone-Loaded Chitosan/Hyaluronic Acid Nanoparticles Suppress Psoriasis by Deactivating STAT3 Pathway and Restricting Immune Cell Recruitment. Asian J. Pharm. Sci..

[114] Zhu Z., Chen J., Lin Y., Zhang C., Li W., Qiao H., Fu M., Dang E., Wang G. (2020). Aryl Hydrocarbon Receptor in Cutaneous Vascular Endothelial Cells Restricts Psoriasis Development by Negatively Regulating Neutrophil Recruitment. J. Investig. Dermatol..

[115] Kim J., He Y., Tormen S., Kleindienst P., Ducoli L., Restivo G., Drach M., Levesque M.P., Navarini A.A., Tacconi C. (2023). The P300/CBP Inhibitor A485 Normalizes Psoriatic Fibroblast Gene Expression In Vitro and Reduces Psoriasis-Like Skin Inflammation In Vivo. J. Investig. Dermatol..

[116] Tsukayama I., Kawakami Y., Tamenobu A., Toda K., Maruoka S., Nagasaki Y., Mori Y., Sawazumi R., Okamoto K., Kanzaki K. (2022). Malabaricone C Derived from Nutmeg Inhibits Arachidonate 5-Lipoxygenase Activity and Ameliorates Psoriasis-like Skin Inflammation in Mice. Free Radic. Biol. Med..

[117] Zhou Y., Han D., Follansbee T., Wu X., Yu S., Wang B., Shi Z., Domocos D.T., Carstens M., Carstens E. (2019). Transient Receptor Potential Ankyrin 1 (TRPA1) Positively Regulates Imiquimod-Induced, Psoriasiform Dermal Inflammation in Mice. J. Cell. Mol. Med..

[118] Okada F., Izutsu R., Goto K., Osaki M. (2021). Inflammation-Related Carcinogenesis: Lessons from Animal Models to Clinical Aspects. Cancers.

